# Host Genotype and Weather Effects on Fusarium Head Blight Severity and Mycotoxin Load in Spring Barley

**DOI:** 10.3390/toxins14020125

**Published:** 2022-02-08

**Authors:** Felix Hoheneder, Eva Maria Biehl, Katharina Hofer, Johannes Petermeier, Jennifer Groth, Markus Herz, Michael Rychlik, Michael Heß, Ralph Hückelhoven

**Affiliations:** 1 Chair of Phytopathology, School of Life Sciences, Freising-Weihenstephan, Technical University of Munich, 85354 Freising, Germany; felix.hoheneder@tum.de (F.H.); katharina.marlene.hofer@gmail.com (K.H.); m.hess@tum.de (M.H.); 2 Chair of Analytical Food Chemistry, School of Life Sciences, Freising-Weihenstephan, Technical University of Munich, 85354 Freising, Germany; eva.biehl@tum.de (E.M.B.); michael.rychlik@tum.de (M.R.); 3 Plant Health and Environment Laboratory, Ministry for Primary Industries, Auckland 1072, New Zealand; 4 Chair of Mathematical Modelling of Biological Systems, Department of Mathematics, Technical University of Munich, 85748 Garching, Germany; hannes.petermeier@tum.de; 5 Institute for Crop Science and Plant Breeding, Bavarian State Research Center for Agriculture (LfL), 85354 Freising, Germany; jennifer.groth@lfl.bayern.de (J.G.); markus.herz@lfl.bayern.de (M.H.); 6 German Environment Agency (Umweltbundesamt), 06844 Dessau, Germany

**Keywords:** fusarium head blight, *F. culmorum*, *F. avenaceum*, spring barley, environment, resistance breeding, deoxynivalenol, enniatins

## Abstract

Epidemiology of Fusarium Head Blight (FHB) of spring barley is relatively little understood. In a five-year study, we assessed quantitative resistance to FHB in an assortment of 17 spring barley genotypes in the field in southern Germany. To this end, we used soil and spray inoculation of plants with *F. culmorum* and *F. avenaceum*. This increased disease pressure and provoked genotypic differentiation. To normalize effects of variable weather conditions across consecutive seasons, we used a disease ranking of the genotypes based on quantification of fungal DNA contents and multiple *Fusarium* toxins in harvested grain. Together, this allowed for assessment of stable quantitative FHB resistance of barley in several genotypes. Fungal DNA contents were positively associated with species-specific *Fusarium* toxins in single years and over several years in plots with soil inoculation. In those plots, plant height limited FHB; however, this was not observed after spray inoculation. A multiple linear regression model of recorded weather parameter and fungal DNA contents over five years identified time periods during the reproductive phase of barley, in which weather strongly influenced fungal colonization measured in mature barley grain. Environmental conditions before heading and late after anthesis showed strongest associations with *F. culmorum* DNA in all genotypes, whereas for *F. avenaceum*, this was less consistent where we observed weather-dependent associations, depending on the genotype. Based on this study, we discuss aspects of practical resistance breeding in barley relevant to improve quantitative resistance to FHB and associated mycotoxin contaminations.

## 1. Introduction

Barley is a relatively stress-resilient crop that can be grown even under unfavourable conditions. It is mainly used as animal feed and, when it comes to spring barley, for producing malt for brewing and food industries. Malting barley is a relatively high-value crop with high demands regarding grain quality. Pests and pathogens are provoking losses in yield and quality and threaten global barley production. Besides foliar diseases, where Ramularia leaf spot (caused by the fungus *Ramularia collo-cygni*) recently became the most dominant disease in barley in many countries worldwide [1], fungi of the genus *Fusarium* directly damage barley spikes. The *Fusarium* pathogen complex comprises several species, which are together the causal agent for Fusarium Head Blight (FHB) in cereal crops, provoking high yield and product quality losses. *F. graminearum*, *F. culmorum*, *F. avenaceum*, *F. sporotrichoides*, *F. poae*, *F.* *langsethiae, F. tricinctum, F. equiseti, F. cerealis* and *F. asiaticum* are the most prevalent species infecting small grain cereals [2,3,4], among several other species according to global regions [5]. *Fusarium* infection of barley heads is associated with a multi-layered, but unspecific, symptomatology regarding multiple grain alterations: (i) discoloration—necrotic spikelets, brown to purple-black spots on spelts, lemma and palea, pale to reddish grain colour; (ii) form—small or aborted grains, shrunken and shrivelled grain texture; (iii) no visible symptoms [3,6,7,8]. In contrast to wheat, premature and blight head sections are rare in barley [4,6]. *Fusarium* spp. grain infections possess harmful mycotoxin contaminations of animal feed and human food. Mostly zearalenone (ZEA) or trichothecens with its prominent representative deoxynivalenol (DON) are contaminants in cereal grains, among several others and their derivates [9,10].

Infected grains cause various technological problems concerning quality and safety of processed barley products. Discoloured grain leads to rejection of barley batches for malting or a downgrade to marketing and in price [8]. *Fusarium*-damaged grains exhibit low germination capacity during malting and further alteration of wort colour [11]. Grain infected with *Fusarium* spp. reveal modulations in gene expression of amylolytic and cytolytic enzymes during malting [12], resulting in difficulties during mashing and subsequent fermentation processes [8,11,12,13]. 

Depending on diverse geographical and meteorological factors, the predominance of several *Fusarium* species is highly variable from global regions to a field scale, between seasons and over longer time periods [14,15,16,17]. On a global scale, the most predominant species associated with FHB is *F. graminearum*. Other related species, e.g., *F. culmorum* and *F. avenaceum,* have high relevance due to their high potential to cause symptoms, yield reductions and considerable loads of mycotoxins in barley grains and malt [7,9,12,18,19]. The sympatric coexistence of several *Fusarium* species enhances plasticity of field populations to adapt to diverse environmental conditions, individual cultivation practises and to different host genotypes (cultivars) [3,5,15,19]. Variable weather conditions over seasons provoke presence of distinct *Fusarium* species with their individual agro-ecological niche, and consequently *Fusarium* infestations and mycotoxin contaminations are highly variable and challenging in control [5,10,20]. However, as recently reported, shifts in composition of populations lead to emergence of new chemotypes with changes in toxicity and aggressiveness worldwide [21], even with a replacement of one chemotype by a more toxigenic one within the same species [22]. Several studies suggest alterations towards dominance of *F. graminearum*, replacing other *Fusarium* species in recent years in Central Europe [17,23] and Northern America [24]. A general increase of *Fusarium* incidence and strong dynamics within the species complex was observed in barley grain over the last decades in Bavaria [17]. In the same time period, rising annual mean temperatures, air humidity and changes in management practises, e.g., expanded maize cultivation, may have enhanced FHB incidence. Other studies suggest similar trends and changes within the species composition towards 2050 in Northern Europe [25], in the UK [26] or even entire Europe [27], where rising temperatures and atmospheric CO_2_ levels will likely increase favourable conditions for FHB infections. It is suggested that warmer conditions will likely increase proportions of soil-borne pathogens and inocula worldwide [28,29]. Moreover, extreme weather events, application of no-tillage practises and intensification of cereal production with narrowed crop rotations could drive FHB outbreaks and mycotoxin contaminations, which will likely become a challenge in the future throughout the whole barley value chain [28,30,31,32]. 

Plant breeding is the most successful tool for a sustained adaptation of crops to adverse climatic conditions, various epidemic pathogens and pests. Potential genetic resources for improvement of barley to future climate scenarios are present [33], but so far they are still not comprehensively utilized and not fully understood in their multiple functions [34]. Hence, breeding FHB-resistant cultivars is challenging in regard to diverse interacting abiotic and biotic factors [35,36]. In particular, it is a necessity that genotypes maintain high pathogen resistance under adverse environmental conditions. Therefore, understanding plant physiology of diverse interactions between genotype, pathogen and environment is crucial for breeding progress, but it is complex because the impact of single factors on host responses is highly specific and mainly not additive [37]. The complexity of FHB resistance results, in general, from the interdependencies of different components: diverse impacts of weather conditions on site and the microclimate on the pathogen and host resistance as well as the accurate identification of resistant breeding material through targeted inoculation trials, appropriate diagnostic tools, selection by comprehensive phenotyping and the use of genetic markers [20,38].

Utilizing genetic resources towards FHB resistance in barley is limited because this trait mostly derives from less adapted and exotic genotypes, which possess poor agronomic characteristics. Furthermore, up to now no major resistance gene effective in protection against FHB was found in wheat and barley germplasm [39,40,41,42,43,44]. Numerous quantitative trait loci (QTL) associated with FHB resistance were identified in barley, but pleiotropic effects on agronomic and physiological traits and linkage of unfavourable traits throughout inheritance make breeding of FHB-resistant barley difficult. Furthermore, the genetic background of an individual candidate has a strong impact on expression of FHB resistance, which produce different disease scores in terms of patho-phenotyping in the field. The latter is strongly biased by the environment, infection pressure in inoculation nurseries, aggressiveness and chemotype of the used *Fusarium* spp. isolates, inoculation method, plant development and morphology. Hence, exploitation of FHB QTL is challenging because resistance appears to be exclusively quantitative in elite barley cultivars and determines the basal level of infection [20,44,45,46]. Additionally, basal FHB resistance is associated with different components (resistance types I to VI) contributing independently to different layers of defence, which are often not equally cumulated or strongly expressed in one genotype. The components were mainly described in wheat, and transfer to barley and other cereal crops is limited [41,47,48,49,50]. In contrast to wheat, barley generally exhibits a strong suppression of fungal growth from the primary infected floret into and through the rachis. This type II resistance is present even in susceptible cultivars, which suggests to further select for type I resistance. Instead, fungal inter-spikelet colonization via the plant surface is a major path for spreading across barley [4,6]. Furthermore, resistance to grain infection and tolerance towards yield loss are probably linked in barley, whereby resistance to trichothecene accumulation possibly acts independently [51], which is a dilemma for efforts to avoid symptomless but mycotoxin-contaminated grain in the entire processing.

The present field study shows a strong differentiation in quantitative FHB resistance within an assortment of German spring barley cultivars and current breeding material over five years. For disease phenotyping, we used soil surface inoculation with bruised grain material colonized with a mixture of different *F. culmorum* or *F. avenaceum* isolates to increase disease pressure and to expose barley heads to a high amount of soil-borne inocula. Quantification of fungal DNA and various *Fusarium* mycotoxins as well as assessment of head symptoms and plant height revealed candidates indicating high basal resistance towards FHB under various weather conditions and seasons. Our results suggest that evaluation of basal FHB resistance identifies suitable genotypes in the field. We further discuss the epidemiology of FHB in barley under aspects of practical breeding.

## 2. Results

### 2.1. Content of Fungal DNA in Grain of Spring Barley Genotypes Inoculated with Fusarium spp. and Evaluation of Basal Quantitative FHB Resistance

We used bruised grain inoculum colonized with *Fusarium* spp. isolates to increase disease pressure from the soil surface and to assess basal resistance towards FHB over five years under variable weather and field conditions. Non-inoculated plots were used to compare disease severity after inoculation with natural infection. Therefore, we extracted genomic DNA from mature barley heads to assess grain infection after inoculation with either *F. culmorum* or *F. avenaceum* isolates, which produce high amounts of distinct types of mycotoxins [44]. We further calculated genotype ranks from DNA and toxin contents for each season and a total mean rank to normalize year effects on *Fusarium* infestation in the field. 

**Figure 1:**
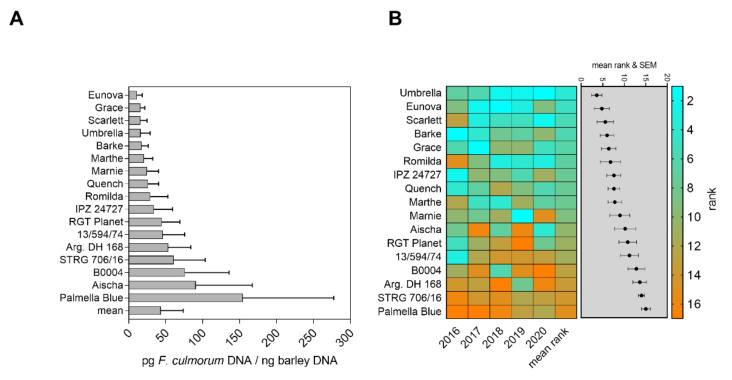
FHB resistance towards *F. culmorum* infection according to DNA contents and mean ranks in field trials between 2016 and 2020. The bar graphs represent mean *F. culmorum* DNA per genotype and total mean in inoculated sub plots in pg *F. culmorum* DNA/ng barley DNA (**A**). The heat map represents scored ranks according to detected *F. culmorum* DNA in mature barley heads in inoculated sub plots. Mean ranks of individual genotypes and the respective standard error of the mean are presented in the grey shaded box (**B**). Low ranks indicate high FHB resistance; high ranks indicate low FHB resistance. Error bars indicate standard error of the mean.

Soil inoculation with bruised grain material revealed a high differentiation in resistance to *F. culmorum* infection over five consecutive years (Figure 1). Mean *F. culmorum* DNA in barley grain ranged from 10.97 to 154.28 pg fungal DNA/ng barley DNA (factor 14.1). The genotype Eunova was most resistant, and Palmella Blue the most susceptible genotype, but also revealed highest variations in grain infestation. Low fungal DNA contents were also detected in Grace, Scarlett, Umbrella and Barke (Figure 1A). To balance variations in infection among single years and to rate FHB resistance based on fungal DNA contents in harvested grain, a year-wise ranking (rank 1 to 17 for each single genotype) and a total mean rank (*n* = 5 years) for individual genotypes were calculated, resulting in a slightly different genotype order compared to the order by average DNA contents. The resulting ranks after inoculation are presented in Figure 1B. Palmella Blue (mean rank 15.0), STRG 706/16 (mean rank 14.0), Argentinische DH 168 (mean rank 13.6), B0004 (mean rank 12.8) and 13/594/74 (mean rank 11.2) ranked highest and can thus be rated as susceptible to *F. culmorum*. Umbrella (mean rank 3.6) and Eunova (mean rank 4.8) reached the lowest mean ranks over five years, indicating environmentally stable and high quantitative resistance despite diverse weather conditions in those years. In addition, the cultivars Scarlett (mean rank 5.6), Barke (mean rank 6.0) and Grace (mean rank 6.4) showed medium ranks and can be judged as moderately resistant to *F. culmorum*. Moderately susceptible genotypes (mean ranks between 7.6 and 10.2) often showed variable ranks among individual seasons. We detected low DNA contents and high year-to-year variability of results in the corresponding non-inoculated plots of all tested genotypes, where total mean of fungal DNA reached only 2.86 pg *F. culmorum* DNA/ng barley DNA (Figure S1). Individual rankings of the non-inoculated plants showed high variations between each season and genotype, indicating a random pattern of natural infection under the given conditions (Figure S1B). We hence consider those data less representative for genotype-dependent FHB resistance, although Palmella Blue was similarly ranked susceptible and Barke resistant.

**Figure 2:**
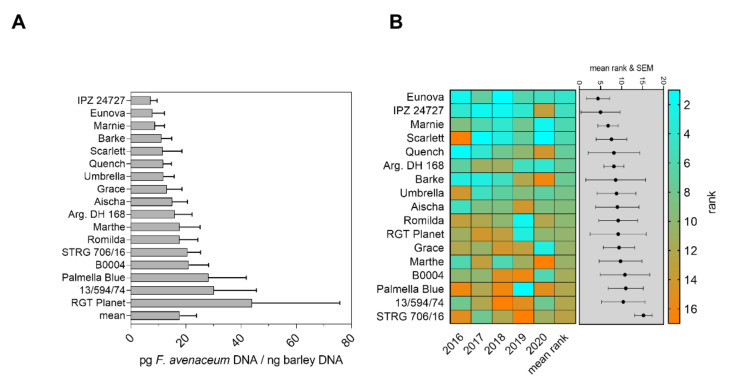
FHB resistance towards *F. avenaceum* infection according to DNA contents and mean ranks in field trials between 2016 and 2020. The bar graphs represent mean *F. avenaceum* DNA per genotype and total mean in inoculated sub plots in pg *F. avenaceum* DNA/ng barley DNA (**A**). The heat map represents scored ranks according to detected *F. avenaceum* DNA in mature barley heads in inoculated sub plots. Mean ranks of individual genotypes and the respective standard error of the mean are presented in the grey shaded box (**B**). Low ranks indicate high FHB resistance; high ranks indicate low FHB resistance. Error bars indicate standard error of the mean.

Contents of *F. avenaceum* DNA in mature grains of soil-inoculated barley genotypes revealed a differentiation in disease severity in the field over five years (Figure 2A). The cultivars IPZ 24727, Eunova and Marnie were least infected by *F. avenaceum* (fungal DNA contents were 7.21, 7.89 and 8.78 pg *F. avenaceum* DNA/ng barley DNA, respectively). Highest amounts of fungal DNA were recorded in grains of RGT Planet, breeding line 13/594/74 and Palmella Blue (43.96, 30.12 and 28.21 pg/ng barley DNA) (Figure 2A). Ranking of disease severity showed a clear differentiation between genotypes, in particular, resistant or susceptible to *F. avenaceum* infection over five years. The genotypes Eunova, IPZ 24747, Marnie and Scarlett scored the lowest ranks indicating environmentally stable quantitative resistance (mean ranks: 4.2, 5.0, 5.8 and 5.8). Despite variations of ranks among individual years, highest mean ranks were calculated for breeding lines STRG 706/16 (12.6), 13/594/74 (12.0) and Palmella Blue (12.0), revealing susceptibility towards *F. avenaceum* under variable conditions in the field (Figure 2B). Natural infection resulted in low *F. avenaceum* DNA contents in a range between 0.82 and 9.81 pg/ng barley DNA. Only Palmella Blue and Umbrella were noticeably infected, but with high variations (Figure S2A). Under naturally infected control conditions, yearly individual ranks of most genotypes varied, suggesting that the natural inoculum was insufficient for a reliable disease phenotyping (Figure S2B).

Taken together, the data reveal an increase in *Fusarium* spp. DNA contents in harvested grains after application of elevated disease pressure. Inoculation resulted in a clear differentiation in the level of quantitative FHB resistance among several barley genotypes, compared to non-inoculated control plants.

We additionally genotyped the 17 barley genotypes by the use of a 9000 single nucleotide polymorphism array to assess their relationship. Hierarchical clustering of informative marker data showed that few genotypes either resistant (Umbrella, IPZ 24727, Barke, Grace) or susceptible (13/594/74, STRG 706/16) to *F. culmorum* or *F. avenaceum,* respectively, clustered together. They could, hence, share a common genetic basis for the observed FHB resistance/susceptibility within the barley assortment (Figure S3).

**Figure 3:**
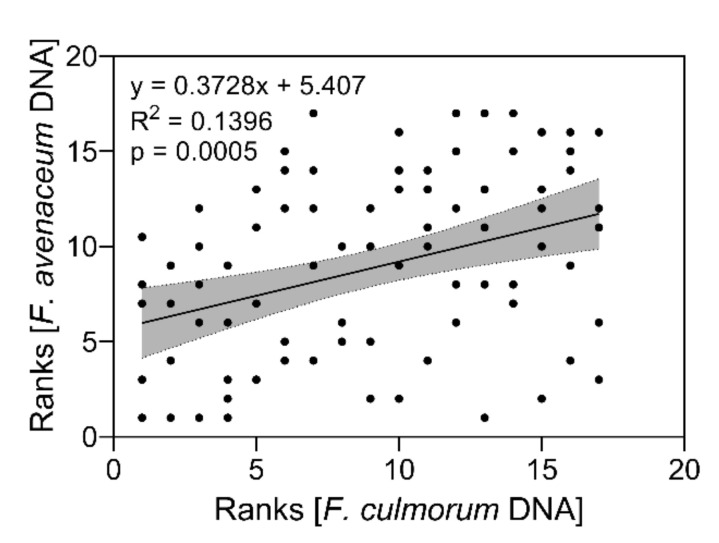
Relation between calculated resistance ranks according to *F. culmorum* and *F. avenaceum* DNA content in mature barley heads after bruised grain inoculation in field trials between 2016 and 2020. Data analysis: Simple linear regression; *p* value indicates significance of the slope from zero. Grey shaded areas represent 95% confidence bands of the best-fit line.

Correlating the ranks of calculated resistance under inoculation with either *F. culmorum* or *F. avenaceum* colonized bruised grain material revealed a significant (*p* < 0.001) relation between ranks of single inoculation trials over five years in the field though with a low R^2^ of 0.1396 (Figure 3). Hence, genotypes with resistance to one *Fusarium* species are likely to be resistant to other *Fusarium* species.

### 2.2. Evaluation of Genotype Resistance and Pathogen Abundance after Fusarium spp. Co-Inoculation

For determination of FHB resistance under co-inoculation, we measured fungal DNA contents in harvested grain after co-inoculation with mixed bruised grain material colonized with *F. culmorum* and *F. avenaceum* in micro field plots in 2017 and 2018. *F. culmorum* reached generally higher DNA contents than *F. avenaceum* in grain material. Many genotypes ranked similarly after co-inoculation, when compared to separate inoculation, and there was a positive correlation between fungal DNA contents and plant disease ranks when comparing both fungal species. Taken together, the data further support that barley genotypes resistant towards one *Fusarium* spp. species are likely to be resistant to other species even when fungi co-occur in the inoculum (Figure S4).

### 2.3. Correlation of Fusarium spp. DNA Contents and Mycotoxin Contamination in Barley Grain

**Figure 4:**
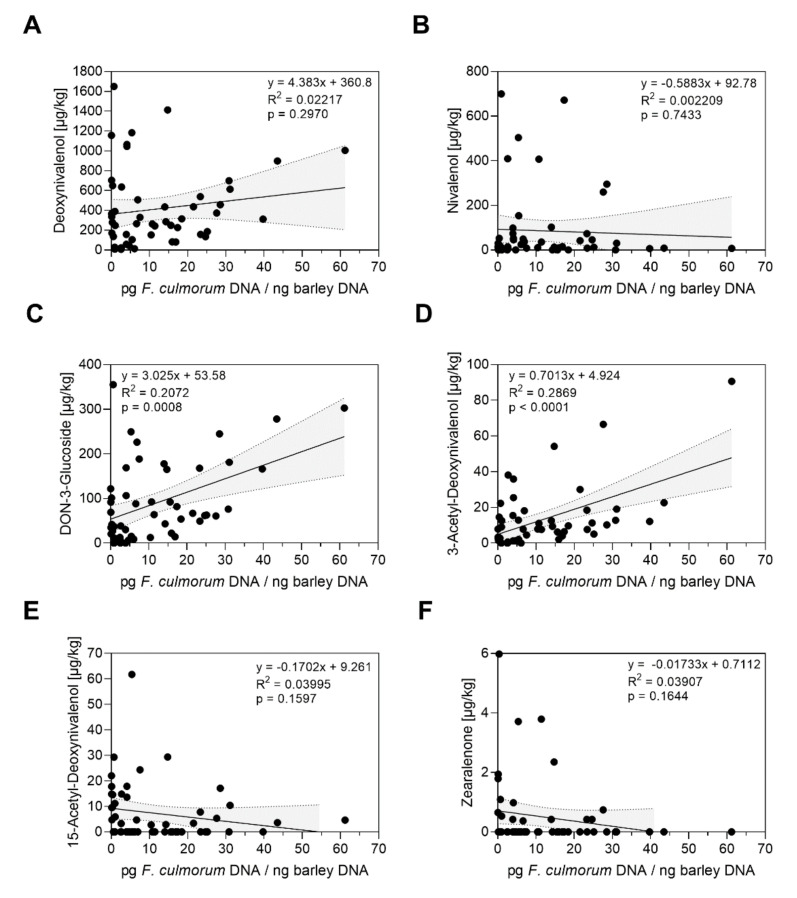
Relations between *F. culmorum* DNA and *Fusarium* toxin contents in mature barley heads after bruised grain inoculation in the field between 2018 and 2020. The figures show contents of *F. culmorum* DNA each correlated with (**A**) contents of deoxynivalenol, (**B**) contents of nivalenol, (**C**) contents of deoxynivalenol-3-glucoside, (**D**) contents of 3-acetyl-deoxynivalenol, (**E**) contents of 15-aceytl-deoxynivalenol and (**F**) contents of zearalenone in µg/kg. Data analysis: Simple linear regression; *p* value indicates significance of the slope from zero. Grey shaded areas represent 95% confidence bands of the best-fit line.

In the context of grain infection and genotype resistance, we further used grain samples of mature barley heads from the bruised grain inoculation trials between 2018 to 2020 to measure multiple *Fusarium* toxins. The data were correlated with fungal DNA contents of the respective sample to evaluate mycotoxin production in relation to fungal colonization over three seasons. Compared to all detected mycotoxins possibly produced by *F. culmorum*, the contents of DON were highest in the grain samples inoculated with *F. culmorum* with a maximum of 1648.64 µg/kg (Figure 4). However, we did not observe a general positive relation of DON contents with *F. culmorum* DNA over three years (*p* = 0.2970; R^2^ = 0.02217) (Figure 4A), but we did observe significant, positive correlations for data of each single year (Table S1). A positive correlation was also observed between *F. culmorum* DNA contents and deoxynivalenol-3-glucosides (DON3G) (*p* = 0.0012; R^2^ = 0.1987) and 3-acetyl-deoxynivalenol (3ADON) (*p* = 0.0001; R^2^ = 0.2753) over three years (Figure 4C,D).

**Figure 5:**
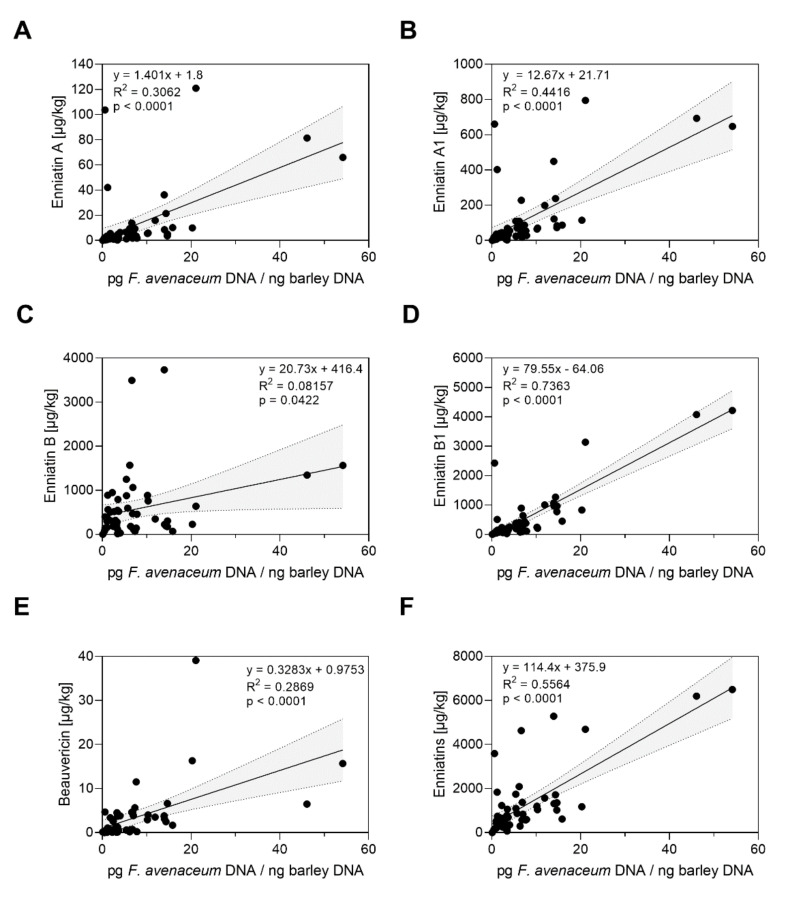
Relations between *F. avenaceum* DNA and *Fusarium* toxin contents in mature barley heads after bruised grain inoculation in the field between 2018 and 2020. The figures show contents of *F. avenaceum* DNA each correlated with (**A**) contents of enniatin A, (**B**) contents of enniatin A1, (**C**) contents of enniatin B, (**D**) contents of enniatin B1, (**E**) contents of beauvericin and (**F**) sum of all detected enniatins in µg/kg. Data analysis: Simple linear regression; *p* value indicates significance of the slope from zero. Grey shaded areas represent 95% confidence bands of the best-fit line.

Furthermore, the detected contents of *F. avenaceum* DNA were significantly positively correlated with contents of enniatins (ENN) A, A1, B and B1, respectively, total sum of all detected enniatins and beauvericin (Figure 5).

**Figure 6:**
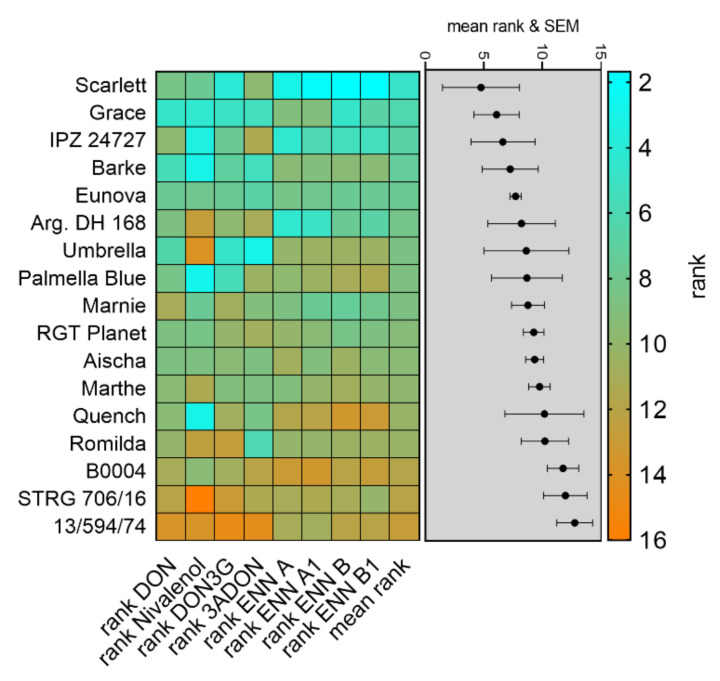
Genotype ranking according to detected *Fusarium* toxin contents in mature barley heads after bruised grain inoculation in the seasons 2018 to 2020. The heat map represents scored mean ranks according to detected contents of DON, NIV, DON3G, 3ADON, enniatins A, A1, B and B1 in mature barley heads in inoculated sub plots. Mean ranks of individual genotypes and the respective standard error of the mean are presented in the grey shaded box. Low ranks indicate high FHB resistance; high ranks indicate low FHB resistance. Error bars indicate standard error of the mean.

Mean contents of the most predominant *Fusarium* toxins produced by *F. culmorum* or *F. avenaceum* were used to calculate severity ranks for mycotoxin contamination in the same manner as for fungal DNA contents (Figure 6). The data reveal a low mean rank for Scarlett (mean rank 4.75), indicating little contamination with *Fusarium* toxins over three years. In contrast, genotype 13/594/74 had high ranks for DON, NIV, DON3G, 3ADON and enniatins resulting in the highest mean rank (12.75) equal to strong contaminations with mycotoxins and indicating low resistance towards mycotoxin production after *Fusarium* infection (Figure 6).

### 2.4. Correlation of Plant Height and Grain Infection

**Figure 7:**
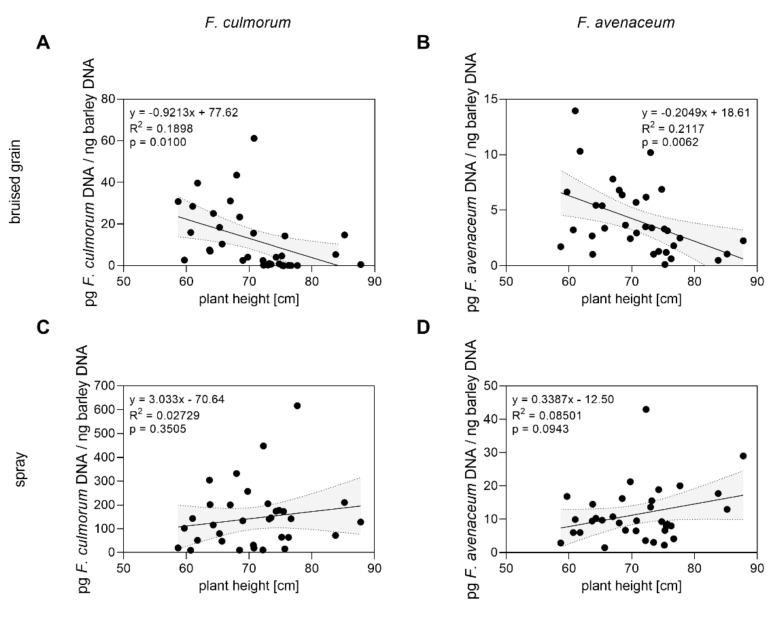
Relations between mean plant height and mean *F. culmorum* or mean *F. avenaceum* DNA in mature barley heads in field trials between 2019 and 2020. Data points represent data of 17 genotypes in bruised-grain-inoculated (**A**,**B**) and in spray-inoculated (**C**,**D**) plots, respectively, and mean plant height of each genotype recorded in 2019 and 2020. Data analysis: Simple linear regression; *p* value indicates significance of the slope from zero. Grey shaded areas represent 95% confidence bands of the best-fit line.

Parallel to inoculations with *Fusarium*-colonized bruised grain, we conducted spray inoculations in 2019 and 2020 to evaluate genotype resistance according to different inoculation methods and to evaluate plant architecture-related effects on FHB. Therefore, we measured individual plant height in the field, which can be associated with a passive resistance in terms of spreading of fungal inoculum from the ground up to the heads. Therefore, we used the obtained data to assess relations between plant height and infection of plants inoculated with *Fusarium*-colonized bruised grain or after direct spray inoculation of barley heads, which was conducted in parallel in years 2019 and 2020. Assuming linearity, there was a significant (*p* < 0.05), negative correlation between plant height and *F. culmorum* (*p* = 0.01; R^2^ = 0.1898) or *F. avenaceum* DNA (*p* = 0.0062; R^2^ = 0.2117), respectively (Figure 7), in harvested barley grains from bruised grain-inoculated plots (Figure 7A,B). No correlations (*p* > 0.05) between plant height and *F. culmorum* or *F. avenaceum* DNA, respectively, were found for plants which were spray-inoculated (Figure 7C,D) or naturally infected (Figure S5). The results suggest that, indeed, barley plant height might constitute a hurdle for soil-borne inoculum on its way to ear infection.

### 2.5. Genotype Resistance under Different Inoculation Methods and Comparison of Bruised Grain Inoculation with Spray Inoculation

In order to compare genotype-specific resistance under different inoculation methods, we conducted spray inoculations in 2019 and 2020 in parallel to inoculation via the soil surface with bruised grain material. Spraying of heads with *Fusarium* spp. spore solutions resulted in elevated fungal DNA contents compared to bruised grain inoculation among all tested genotypes (Figure S6A,B). Average *F. culmorum* DNA was by far higher after spray inoculation than after bruised grain inoculation (Figure S6A). Romilda, Quench and Aischa were the most infected genotypes after spray inoculation. *F. culmorum* DNA was lowest in Grace and Marnie after spray inoculation (Figure S6A). Under spray inoculation with *F. avenaceum*, the cultivars Quench and RGT Planet were strongest infected, while Eunova and Scarlett were least infected (Figure S6B). Correlation between both inoculation methods generally did not show a relation between fungal DNA contents (Figure S6C,D). Nevertheless, ranks for *F. avenaceum* contents positively correlated between differently inoculated plots (Figure S6D).

### 2.6. Correlation of Head Symptoms and Fungal Grain Infestation

In order to evaluate a relation between fungal DNA contents in harvested barley grain and head symptoms, a head symptoms index was calculated from disease severity, and proportions of symptomatic heads (incidence) were visually assessed in the field. Head symptoms and DNA contents positively correlated for plants spray-inoculated with *F. culmorum* or *F. avenaceum*, respectively, and for *F. avenaceum* in mock-inoculated plots (Figure S7C,D). However, disease phenotyping based on FHB symptoms appears insufficient to rate barley resistance to *Fusarium* head infection because after successful inoculation with bruised grains that partially resulted in high fungal DNA contents, symptoms did not generally appear (Figure S8).

### 2.7. Effect of Weather on Yearly Infection Success

**Figure 8:**
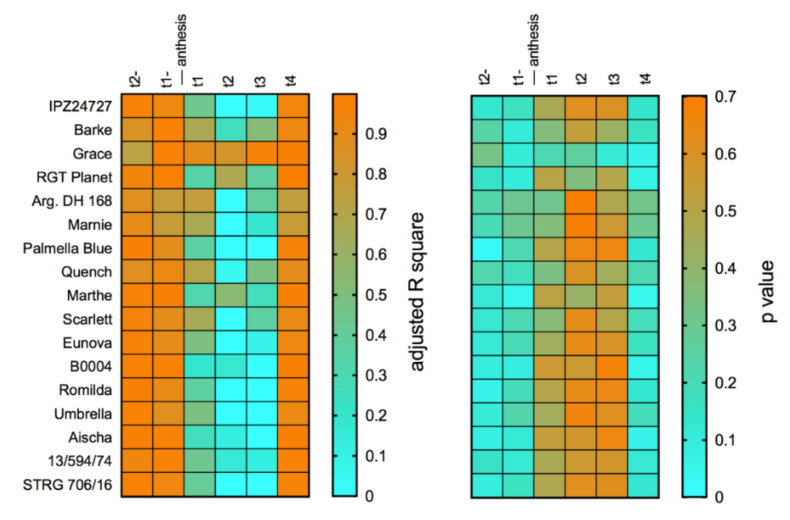
Adjusted R^2^ and respective *p* values of genotype-wise multiple linear regression models for the two weeks before and four weeks after anthesis relative to flowering date in the seasons 2016 to 2020. The model Equation (1) was used to calculate multiple linear regressions to determine effects of weather conditions before and after anthesis on content of *F. culmorum* DNA in mature barley heads.

In addition to disease monitoring in the field, we further recorded weather conditions for a better interpretation of conditions or time frames that support head infection. Therefore, weather data were collected from a nearby weather station during seasons 2016 to 2020 (Figure S9). Weekly sum of temperature, sum of precipitation and mean relative air humidity, respectively, were calculated for the two weeks before and for the four weeks after anthesis in each individual season (Tables S2–S4). We used data of *Fusarium* DNA contents (Figure 1 and Figure 2) and local weather data to set up multiple linear regression models (MLR) to test genotype-wise the influence of weather parameters on FHB severity before and during reproductive phases of barley. According to the selected model (Y(mean DNA content) = β_0_ + β_1_T_SUM_(*week* *x*) + β_2_(T_SUM_(*week x*) × P_SUM_(*week x*)) + β_3_(T_SUM_(*week x*) × RH_AVG_(*week x*)), high associations between *F. culmorum* DNA contents and weather conditions were found for all 17 genotypes in the two weeks before and the fourth week after anthesis (Figure 8). For these time periods, adjusted R^2^ values were in a range of 0.7292 to 0.9985. Corresponding *p* values were accordingly low (*p* < 0.05) during these time periods. In contrast, comparably lower adjusted R^2^ values were found for the three weeks directly after anthesis (adjusted R^2^ < 0.7577), except for Grace, where we found high associations over all six weeks (adjusted R^2^ between 0.8861 and 0.9705). To sum this up, the MLR model revealed a possible relationship of distinct weather conditions and severity of *F. culmorum* infections in the field, which is possibly independent from the genotype, but strongly dependent on weather at certain periods before anthesis and later during the grain filling stage. Repeating this kind of MLR analysis with *F. avenaceum* DNA data revealed differences in goodness of fit of the selected model (adjusted R^2^) according to considered time periods and between different genotypes (Figure S10). The MLR revealed for most of the tested genotypes high adjusted R^2^ values for the second week before anthesis (adjusted R^2^ > 0.7324, except for Barke, Palmella Blue, Quench, Scarlett) and one week after anthesis (adjusted R^2^ > 0.7082, except for Grace, Palmella Blue, Quench and breeding line 13/594/74). Low adjusted R^2^ values were found for several genotypes (e.g., IPZ 24727, Barke, Grace, Argentinische DH 168) for the week before anthesis and for the second and fourth week after anthesis.

## 3. Discussion

Fusarium head blight in wheat and barley is a major disease worldwide and provokes high losses of yield and grain quality. The exclusively incomplete basal resistance of barley to FHB and the lack of major resistance genes point out the necessity to select diverse genotypes with quantitative resistance crucial to improve resistance breeding. The aim of the present field study was to evaluate an assortment of current breeding material of German barley breeders and spring barley cultivars commonly grown in Europe for quantitative FHB resistance over several seasons and under variable weather conditions. Furthermore, we used different *Fusarium* species and inoculation methods to examine and compare important aspects for selecting candidate genotypes. Our data reveal strong differences in quantitative resistance to FHB between different genotypes and show stable resistance/susceptibility across variable weather conditions in the field. This validates some of the applied methods and eases decision-making in genotype selection in future breeding of modern barley cultivars for stable performance in the field and reduced mycotoxin contaminations of barley products.

In our inoculation trials using *Fusarium* colonized bruised grain material, we successfully increased disease pressure from the soil to provoke a clear differentiation in quantitative FHB resistance of diverse spring barley genotypes over five consecutive seasons (Figure 1 and Figure 2). In particular, the selected inoculation method simulates soil-borne fungal inoculum in the field, which is a major source of infections when spores are transported from the soil surface to the head via rain splash dispersal [2,52,53]. Furthermore, this inoculation technique was proven suitable to study different types of FHB resistance of cereal crops [54].

In addition, quantification of fungal DNA contents appears as a very sensitive tool to quantitatively estimate fungal colonization of barley grain with commonly unspecific or absent head blight and grain symptoms in barley [55]. However, absolute DNA contents varied between single years, most likely due to environmental conditions. Hence, differentiation in genotype resistance was in particular confirmed by repeated field seasons and genotype ranking. To balance year effects in FHB severity over the five years, we ranked DNA contents for each individual genotype and each year and further calculated a total average rank to differentiate between the most susceptible and the most resistant genotypes (Figure 1B and Figure 2B). The disease ranking takes the infection level of all tested genotypes for each season into account. The total mean of disease ranking reduces bias of annual effects on infection and is recommended to display differentiation of genotypes within the tested genotype assortment over several seasons or several environments. Furthermore, different disease parameters can be merged together to one mean rank, which was previously applied for selection of barley genotypes resistant to *Ramularia collo-cygni* in diverse environments in the field [56].

The genotypes Eunova, Grace, Scarlett, Umbrella and Barke revealed lowest contents of *F. culmorum* DNA (Figure 1A), whereby the genotypes Eunova, IPZ 24727 and Marnie were least infected with *F. avenaceum* (Figure 2A). These genotypes further showed very uniform and reproducible disease resistance ranks across the five seasons (Figure 1B and Figure 2B), indicating robust levels of quantitative FHB resistance. In addition, these genotypes by trend genetically cluster together, raising the possibility of a common genetic basis for quantitative FHB resistance (Figure S3). Furthermore, IPZ 24727, Umbrella, Grace, Eunova and Marnie were also moderately to highly resistant to *Ramularia collo-cygni* leaf spots over three years [56] and hence may represent suitable germplasm for further resistance breeding.

Our data could also identify genotypes susceptible to both *Fusarium* species. According to DNA contents and disease rankings, the landrace Palmella Blue and breeding line STRG 706/16 revealed high susceptibility against FHB among other genotypes, which were variably affected by one of the two *Fusarium* species. We further found a general positive relation between disease resistance ranks for both *Fusarium* species (Figure 3). This indicates that genotypes very resistant/susceptible to one *Fusarium* species are likely to be also similarly resistant/susceptible to the other *Fusarium* species and possibly against their associated mycotoxins. However, we only used two different *Fusarium* species, and the data do not represent similar effects between other *Fusarium* species (e.g., *F. graminearum*) commonly infecting barley, which needs to be proven in further experiments using various *Fusarium* species. Within the *Fusarium* complex in cereal grains [2], barley is vulnerable to several *Fusarium* species simultaneously present in the field filling their individual ecological niche, having different pathogen biology and producing an individual spectrum of mycotoxins [7,17,57]. However, [58] found that resistance of wheat genotypes against *F. graminearum* was similar to resistance to other *Fusarium* species. Similar studies on barley using different *Fusarium* species revealed variable resistance to differently pathogenic isolates and *Fusarium* species, suggesting selection to strong FHB resistance is only conclusive when various species and isolates are used for inoculation trials [59]. For our inoculation trials, we used different species and isolates, respectively, which were shown to be pathogenic on barley in previous field trials and greenhouse studies [7,12,44].

Comparing DNA contents and genotype ranks of artificially inoculated plants with naturally infected plants, we observed very high variations of DNA contents in barley grain and further disease rankings (Figures S1 and S2). This suggests that natural infection randomly occurred across individual years and field plots and is not sufficient to provoke reproducible differentiation in disease resistance. Associations between FHB disease parameters of barley genotypes under natural infection and artificial inoculation are difficult to assess and were only found for low DON concentrations in one study by [46]. According to our data and from other studies on FHB resistance in barley, artificial inoculation highlights genotypic differences when the inoculum dose and pathogenic isolates are well adjusted to the chosen inoculation method to reproduce increased disease pressure and genotypic differentiation in a range, which is not overwhelming genotypic resistance [40,59,60,61]. This can be obtained in barley either via inoculation of the soil or by spray inoculation. Point inoculation of spikes showing type II resistance is more recommendable for selecting FHB resistance in wheat, but not in barley, where strong type II resistance is common, and type I resistance is more decisive for high quantitative resistance [60,61]. We conclude from our data on soil and spray inoculation, in comparison to natural infection, that only artificial inoculation provokes environmentally stable and sufficient disease pressure for genotype selection in front of the strong influence of weather conditions. This is in line with previous studies [40,59,60] and in contrast to results from [46]. We suggest that genotype selection is less conclusive without artificially increased disease pressure because the local dose of natural inoculum cannot be controlled and occurs randomly in the field.

Considering aspects of morphological characteristics such as plant architecture and height, we compared FHB severity dependent on the inoculation method and genotype-specific plant height. Our data show a negative relation between height and *F. culmorum* or *F. avenaceum* DNA contents after soil surface inoculation with bruised grain material. No such relation was found in spray inoculated plants (Figure 7). This supports the finding that plant height is a hurdle for transport of spores from the soil up to the head, decreasing contact of spores with the head and lowering initial infection, resulting in possibly less FHB severity [52,53,62]. However, data on plant height associated FHB resistance are still rare for barley, and probably more complex than for wheat, because the slope of the head and presence of awns, for example, represent barley-specific factors that may affect spore contact differently than in wheat. Hence, our data suggest that taller plants can partially be associated with higher resistance in a passive way. Our data are also in partial agreement with earlier findings of [63], who also described a negative correlation between plant height and either FHB or mycotoxin content in spring barley after spray inoculation across two years. However, we observed this only after soil inoculation for DNA contents, and we found no correlation between plant height and mycotoxin contents (Tables S1 and S5). According to the literature, several QTLs associated with FHB resistance were described and found to be either linked with plant height [40,64] or independent from height [65], suggesting pleiotropic effects between FHB resistance QTLs and plant height dependent on genetic background of individual barley genotypes/accessions and environmental factors. Summarizing this, basal resistance in relation to plant height should be taken into account during selection of candidate genotypes to assess possible linkage between FHB resistance and plant characteristics.

In 2018, 2019 and 2020 we further quantified *Fusarium* toxin contaminations. The data clearly detected positive correlations of several *Fusarium* toxins and fungal DNA contents (Figure 4 and Figure 5, Table S1). Our data indicate a general relation between fungal colonization and contents of multiple but species-specific *Fusarium* toxins in single years (Table S1) and over three individual seasons (Figure 4 and Figure 5). This was most clear for toxins usually produced by *F. avenaceum*. Contents of the most predominant *Fusarium* toxin deoxynivalenol were in a positive relation with *F. culmorum* DNA when considering individual seasons (Table S1), but not over three seasons (Figure 4). No correlation was found for nivalenol (Figure 4, Table S1). We did not quantify DNA of *F. graminearum* in grain samples, so we cannot exclude possible contaminations of DON and NIV in *F. culmorum* inoculated plots by additional natural infections by *F. graminearum* at the field site. Hence, relation between *F. culmorum* DNA and DON or NIV contents, respectively, was possibly limited. Taken together, the positive relations between DNA and content of several *Fusarium* toxins over individual seasons with variable environmental conditions demonstrates that quantification of DNA is valid to estimate basal resistance, which appears linked to restricted mycotoxin contamination. This is important when costly and laborious toxin measurements are not manageable, e.g., for plenty of breeding lines. On the other hand, novel methods for simultaneous detection of multiple mycotoxins are available and most promising to collect data during breeding and genotype selection process, and they may detect resistance or susceptibility against selected *Fusarium* species and the accumulation of their associated mycotoxins [66,67]. If possible, it seems recommendable to generate data on both fungal DNA contents of several *Fusarium* species and contents of multiple mycotoxins to gain mutual validity in evaluation of FHB resistance. 

Transformation of mycotoxin contents to a ranking revealed that resistance of individual genotypes to accumulation of toxins produced by *F. culmorum* or *F. avenaceum*, respectively, differentiated similarly as for data on DNA contents in mature grain (Figure 6). The genotypes Grace and Scarlett were rated low and thus show high resistance to fungal colonization (DNA contents) and accumulation to different *Fusarium* toxins, which underscores a possible linkage between different aspects of resistance. These genotypes possibly exhibit strong resistance to initial infection, fungal spreading and an associated resistance to mycotoxin accumulation [68]. Possibly, a machinery for detoxifying mycotoxins even contributes to type I resistance [69,70]. Only a few genotypes (e.g., Quench), revealing moderate rankings, did not generally show low ranks according to mycotoxin contents from both *Fusarium* species, suggesting that only genotypes with a strong basal resistance probably exhibit a broad resistance to several *Fusarium* toxins. On the other hand, we further could detect genotypes (breeding line 13/594/74 and STRG 706/16) that were susceptible to both *Fusarium* species and were also rated high (susceptible) for mycotoxin loads associated with *F. culmorum* or *F. avenaceum*, respectively. Taken together, our year-specific ranking method allows the evaluation of resistant genotypes to different disease parameters using phenotypic data and identifies candidate genotypes for further FHB resistance breeding efforts.

In our field trials, spray inoculation resulted in higher DNA contents in mature barley heads compared to the bruised grain inoculation method (Figure 7 and Figure S6). This clear difference was revealed across all tested genotypes with highly elevated DNA contents of *F. culmorum* (Figure S6). Furthermore, we observed again a clear differentiation in head infection, which enables selection of resistant genotypes after spray inoculation. In comparison to bruised grain inoculation trials, we could detect a few genotypes that showed high FHB resistance after spray inoculation. The genotypes Grace and Marnie showed resistance to *F. culmorum*, and the cultivar Eunova was little infected by *F. avenaceum*. On the other hand, we want to stress that several genotypes judged as moderately or even highly resistant to FHB under bruised grain inoculation over five years showed high susceptibility after spray inoculation (e.g., Umbrella, Romilda and Quench). Furthermore, there was no correlation between resulting DNA contents of *Fusarium* species when we compare both inoculation methods. When we ranked DNA contents of either inoculation method, we obtained a positive correlation for inoculations with *F. avenaceum*. This was not the case for *F. culmorum*. We assume that high doses of aggressive *F. culmorum* spores applied on the head may have overwhelmed quantitative resistance of some genotypes. Bruised grain inoculation, although not suitable to test specifically for type I resistance, may be recommendable when testing genotypes for the overall complex field resistance to FHB. However, direct application of spore suspension could further ease evaluation of resistance against *Fusarium* toxin accumulation in relation to inoculum dose (spore concentration) and fungal biomass in mature barley grain (DNA contents).

Severity of FHB on barley is highly dependent on environmental conditions. Barley is most sensitive to *Fusarium* spp. infection during productive stages around full to late anthesis, especially when conditions are humid and temperatures mild to warm [7,9,16,71]. FHB resistance is further associated with flowering type of barley, which can shift sensitive stages among individual genotypes between full to late anthesis [71]. Furthermore, it was reported that optimal infection conditions of *F. graminearum* are different from those described in wheat and further dependent on the genotype [16]. Hence, studies on climatic conditions on FHB in wheat are less transferable to barley but are crucial to forecast possible infection events in the field and to improve plant protection measurements [72,73]. Therefore, we recorded weather parameters before and post flowering relative to date of full anthesis over five consecutive seasons. Warm and dry conditions in 2017 and 2019 alternated with cool and wet conditions in 2016 and 2020 (Figure 1, Figure 2 and Figure S9, Tables S2–S4). Noteworthy, we could detect barley candidates with stable FHB resistance despite strong year-to-year variations of the weather conditions. 

To improve the understanding of the weather dependency of FHB in barley, we used the collected weather data (Tables S2–S4, Figure S9) and contents of fungal DNA in mature barley heads (Figure 1 and Figure 2) to calculate multiple linear regression models. Therefore, we set up a regression model to estimate the influence of different weather parameters on fungal colonization in a time-dependent manner before and after anthesis (Figure 8 and Figure S10). The most important climatic factors such as temperature, air humidity and precipitation, which directly influence the host plant, pathogen growth and fungal spread in the field, were used as explanatory variables in the model. Furthermore, the model was set to detect distinct time periods, which probably reflect distinct phases of susceptibility of the tested genotypes against FHB over several seasons. Interestingly, the chosen model indicated the environmental conditions that occurred in the two weeks before and the fourth week post anthesis had the highest influence on infection of *F. culmorum* across all tested barley genotypes. Possibly, temperature in combination with sufficient rain and relative air humidity before flowering supported growth and sporulation of *F. culmorum*. It is known that rain events before anthesis may also contribute to growth of mycelia, spore emergence and dispersal of sufficient amounts of spores to upper leaves and in the area of the emerging head [52,53]. This is confirmed by results of [74], where an increase in airborne inoculum of *F. avenaceum* and *F. graminearum* was observed after rain periods. According to our model, the given environmental conditions in the fourth week post flowering possibly had high influence on fungal biomass of barley. During this time period, we found highest means for sum of temperature, sum of precipitation and relative air humidity, representing favourable conditions for fungal growth. At this point, it should be noted that it remains unclear whether initial infection of the flowering spikelets or hyphal growth and cell penetration within tissue of a previously infected and developing grain is the most crucial factor determining fungal biomass in the mature grain. We can only speculate on how weather conditions influence the interplay of seed ripening, activity of the fungus within grain tissue and eventual total grain damage. Our model found low associations of fungal biomass with weather conditions in a three-week period after flowering, and at this stage, barley genotype resistance could be more decisive than environmental conditions. Assuming successful infection during or shortly after full anthesis, different components of genotype resistance could probably keep further colonization at a low level, delaying the fungus to completely damage the infected grain. Then, at later stages of maturity and during ripening, weakened genotype resistance and moist conditions could gain fungal growth late in the season and before harvest, especially when the grain is, e.g., remoistened by late rain events. Alternatively, genotype-specific flowering type, extrusion of anthers and spike characteristics could probably shift sensitivity of barley against head infection to later time periods post flowering [71], demonstrating that critical time points for infection and mycotoxin accumulation are not generally at full anthesis, and such characteristics may contribute to passive resistance. This aspect probably needs to be taken more into account for evaluation of FHB resistance in barley. Possibly, the impact of climatic conditions after flowering on genotype-dependent resistance and progression of fungal colonization needs more attention.

However, the same regression model using data on *F. avenaceum* DNA did not show similarly clear results as for *F. culmorum* DNA (Figure S10), indicating that environmental conditions differently promote infection levels of *Fusarium* species within the entire *Fusarium* complex. Alternatively, random errors due to generally lower infection success of *F. avenaceum* caused a more sensitive interplay of genotypes and weather conditions. In a survey on barley by [75], seasonal and genotype effects on abundance of distinct *Fusarium* species in the field were most significant, demonstrating high complexity in FHB complex composition over several seasons, due to a strong interaction of weather parameters with barley genotypes. Despite seasonal effects, infection of a certain barley genotype was associated with infection of distinct *Fusarium* species over three years.

According to the defined model, environmental conditions during the considered six weeks before and after anthesis of barley could probably explain final *F. avenaceum* DNA contents within grain in a more genotype-dependent manner. High associations were found between weather conditions and DNA contents for seven genotypes. Across most genotypes, the second week before and the first week after anthesis revealed high associations between weather conditions and fungal biomass. Conditions during these two periods revealed mild temperatures and sufficiently high relative air humidity, but low precipitation, compared to the other considered time periods, indicating that rainfalls were probably not the most decisive factor for propagation and infection of *F. avenaceum*. This could be explained by morphological characteristics of the spores of *F. avenaceum*, which are larger and have a shorter-range dispersal compared to *F. culmorum* spores [52]. Furthermore, high abundance and severity of *F. avenaceum* is associated with cool and mild conditions, e.g., in Central and Northern European regions, which probably favours this species [25,76,77].

Taken together, the defined model suggests that relations between environmental conditions and *F. avenaceum* infestation are likely to be partially dependent on the genotype. However, this has to be taken with care based on comparably high *p*-values. *F. culmorum* infestation can be clearly associated with warm and wet conditions at distinct time points during the reproductive phase of barley, and this is observed for all genotypes. Indeed, our data on studying FHB epidemiology in relation to variable weather conditions are relevant to better understand environment-dependent FHB development in spring barley.

## 4. Conclusions

Quantitative FHB resistance of barley is still little exploited and not fully understood. This may be due to a complex interaction among individual genotypes, multiple *Fusarium* species, and strong associations of the *Fusarium* epidemiology with environmental conditions. The present study identified spring barley genotypes with an environmental-stable resistance or susceptibility to FHB after artificial inoculation under field conditions. Our investigations support the need of artificial inoculation for reproducible disease phenotyping and genotype selection. Using disease rankings in addition to absolute fungal DNA contents further allowed for finding genotypes with strong resistance against two *Fusarium* species and to various mycotoxins associated with those species, suggesting that strong quantitative resistance to one *Fusarium* species could probably indicate sufficient resistance to other species. Furthermore, we found associations between weather conditions during the reproductive phase of barley and final grain infection, demonstrating complexity of *Fusarium* epidemiology in barley. Our findings are of possible use for practical resistance breeding and might support future prediction modelling of climate-sensitive and weather-dependent FHB in barley.

## 5. Materials and Methods

### 5.1. Inoculation Trials

In the present study, we evaluated resistance of two-rowed spring barley genotypes to Fusarium head blight in the field. The inoculation trials were conducted in Freising-Weihenstephan (48°23′58.2” N, 11°42′51.5” E; 450 m above sea level; Southern Germany) between March and August in the years 2016 to 2020. Soil type is loamy-silt, and a plough was used for basic soil management combined with power harrow and Cambridge-roll for seedbed preparation. In 2016, 2017 and 2018 an assortment of 59 spring barley genotypes was sown in micro plots consisting of two rows of barley, each with a length of 1 m. The set of genotypes was sown in four blocks consisting each genotype once in a fully randomized design. In 2018, the 59 genotypes were additionally sown in standard field plots with a size of 1.5 × 7.5 m in two replicates. For the following seasons in 2019 and 2020, the assortment was narrowed down to 17 candidate genotypes from which 15 genotypes were selected for stable yield and grain proportions, homogenous heading date and plant height, moderate resistance to Ramularia leaf spot and other foliar diseases and further high performance under different field conditions as previously described by [56]. The additional two genotypes were the frequently used malting barley cultivar Marthe and the FHB-susceptible land race Palmella Blue. In the seasons 2019 and 2020, the 17 genotypes were exclusively sown in randomized standard field plots. For reasons of consistency, only results of these 17 genotypes for all the five seasons are presented throughout the paper.

Foliar diseases were controlled by a single cover-spray with the fungicide Prosaro (Bayer Crop Science) (0.8l/ha) at growth stage 32. Applications of nitrogen fertilizer (80–100 kg/ha calcium ammonium nitrate, CAN) and herbicides were conducted according to standard agricultural practise. No growth regulators were used. After full grain maturity (growth stage (GS) 99), micro plots were harvested by hand and threshed manually with a stand combine harvester; field plots were harvested with a single-plot combine harvester. Dates of sowing, recorded growth stages (GS) and date of harvest of each season are listed in Table S6.

### 5.2. Preparation of Fungal Inoculum

Single spore isolates of *F. culmorum* (Fc002, Fc03, Fc06) and *F. avenaceum* (Fa002, Fa02, Fa04) were obtained from long-term storage (−70 °C) glycerol stocks (culture collection, Chair of Phytopathology, TUM) and further used for preparation of fungal inoculum. Therefore, the isolates were cultivated on ¼ strength PDA media (9.75 g/L potato dextrose agar, 11.75 g/L agar) for 14 days in an incubator (SAN-MLR-351H, SANYO, Munich, Germany) at 20 °C, 70% relative humidity and a 12 h light/dark cycle with additional UV-light (cool white: Osram Lumilux 840; UV-light: Philips TL-D BLB black light blue). The *Fusarium* isolates were propagated on double-autoclaved bruised grain as described by [44]. Briefly, the grain was inoculated with *Fusarium*-colonized agar plugs and incubated in transparent plastic bags sealed with a cotton stopper, covered with aluminium foil under UV light (12 h light/dark cycle) at 16 °C for several weeks. The grain substrate was kneaded every second day to maintain uniform fungal colonization. Each plastic bag contained 150 g of colonized grain substrate and was stored in the dark at 4 °C until use. Before application in the field, bruised grain colonized with different *Fusarium* isolates was manually mixed in equal parts.

Spore solutions were obtained from single isolates of *F. culmorum* and *F. avenaceum* propagated on ¼ strength PDA media as described above. The isolates were cultivated on ¼ PDA until emergence of conidia. Subsequently, mycelium and conidia were scraped off with a microscope slide and suspended in sterile tap water. Spores were separated from mycelium fragments by filtering through four layers of sterile gauze bandage placed in a Büchner funnel. Spore concentration was determined with a haemocytometer and adjusted to 25 × 10^4^ conidia/mL by adding autoclaved tap water. A total of 1 mL/L Tween 80 was added for better wetting on head surfaces. The spore solution was stored in the dark at 4 °C for a maximum of one week.

### 5.3. Inoculation of Barley Plants with Bruised Grain Material and Spraying of Heads

Bruised grain inoculation trials were conducted over all five seasons (2016–2020). Randomized micro plots were arranged in four separate blocks and used for soil inoculation. Each block was inoculated with grain material colonized with *F. culmorum*, *F. avenaceum* or a mixture of both. Therefore, 150 g of grain inoculum per square meter was manually and uniformly distributed on the ground between double rows (micro plot) of each genotype. For mixed inoculations, 150 g of each *Fusarium* sp. grain inoculum was used. Control plots remained free of bruised grain inoculum.

In the field plots, an inoculation window of one square meter within one end of each plot was inoculated with bruised grain inoculum of *F. culmorum* or *F. avenaceum*. The opposing end of each plot included a control window of the same size and was not inoculated, thus distribution of spores from the inoculated window was excluded. Inoculations were conducted twice by the use of 75 g of bruised grain inoculum at tillering and at booting stage, respectively. Each window was surrounded by 25 cm of barley canopy at both sides and the end of the plot to exclude boundary effects. Each window was utilized for visual assessments and sampling of heads.

Spray inoculations were conducted in 2019 and 2020. Therefore, randomized field plots were sown in a second block similar as for bruised grain inoculation trials. Each plot included two windows for inoculation and as a control, with a size of one square meter. A total of 150 mL of a fresh spore solution with a concentration of 25 × 10^4^ conidia/mL was sprayed from the top on the heads during early and mid-anthesis (GS 61 to 65). Spray inoculation was conducted in the morning and repeated once, resulting in a final spore density of 75 × 10^6^ conidia/sqm, similar to [44]. Spray inoculation was repeated 5 to 7 days after first inoculation to compensate minor differences in flowering within each plot and between genotypes. Control windows were sprayed with a mock solution (1 mL Tween 80 per litre H_2_O_dest_).

### 5.4. Weather Conditions

Local weather conditions (daily temperature, precipitation and relative air humidity) were collected by a weather station one kilometre away from the field trials in Freising. Weather data were accessed from the web portal of the Bavarian Agro-meteorology service [78].

### 5.5. Statistical Analysis

In order to point out whether and at which time periods the weather conditions before and after anthesis had affected FHB infection of barley in the field, we used multiple linear least-squares regression (MLR) for each single genotype. Therefore, we used DNA contents of mature barley heads over five years as a dependent response variable. To aggregate the daily obtained weather data in the field, we calculated the sum of temperature per week (*T_SUM_*), sum of precipitation per week (*P_SUM_*) and the average relative air humidity per week (*RH_AVG_*) for the two weeks before and the four weeks after anthesis, respectively (Tables S2–S4). Because of variable anthesis over seasons and minor variance in anthesis across the genotype set, we defined a theoretical day of full anthesis (GS 65) of all genotypes as baseline date for each season. Consequently, *T_SUM_*_(*week x*)_, *P_SUM_*_(*week x*)_ and *RH_AVG_*_(*week x*)_ (with × = −2, −1 weeks before and × = 1, 2, 3 or 4 weeks after anthesis) were defined as explanatory variables for multiple linear regression models. MLR was performed using GraphPad Prism version 8.4 for OS X, GraphPad Software, La Jolla California USA.

The model equation for MLR used in this study has the following form:

*Y_(mean DNA content)_ = β_0_ + β_1_T_SUM (week x)_ + β_2_(T_SUM(week x)_ × P_SUM(week x)_) + β_3_(T_SUM(week x)_ × RH_AVG(week x)_)* (1)

Temperature was chosen as the main climatic factor with the most significant impact on infection conditions of barley, as previously described in the literature [7,16]. In addition to sum of temperature (*T_SUM_*_(*week x*)_), the two-way interactions *T_SUM_*_(*week x*)_ with *P_SUM_*_(*week x*)_ and *T_SUM_*_(*week x*)_ with *RH_AVG_*_(*week x*)_ were added to the model. Beforehand, model selection resulted in the shown model in terms of adequate multiple correlation coefficients, high simplicity in context of amount of chosen variables (weather parameter), respective interactions and additive associations of variables. Possible intertwined collinear variables in MLR were previously checked for correlation of climatic parameters by means of scatter plots.

In the current context the intercept *β*_0_ is the mean DNA content without influence of the explanatory variables, whereas from the regression coefficients *β_i_* the change of the dependent variable due to a change in the independent predictor variable can be read. Normality of residuals was tested with the Shapiro–Wilk test. Furthermore, we calculated 95% confidence intervals of *β*_0_ (intercept) and each single *β_i_* to compare them with zero to illustrate their spreading and potential significant effects on the model output for each genotype (Figures S11–S16).

### 5.6. Determination of Plant Height

Plant height was determined with a pocket ruler during anthesis (GS 61 to 67) in 2019 and 2020. Plant height was measured at three spots within each field plot. Average plant height was calculated as the total mean for each genotype.

### 5.7. Assessment of Symptoms and Sampling of Barley Heads

Head symptoms were visually assessed at growth stages 79 to 85. Therefore, anomalies of husks or the entire spikelets, e.g., brown lesions, necrotic spots, brown discolorations and shrivelled grain, were examined throughout the entire inoculation windows. Disease incidence (percentage of symptomatic heads) and disease severity (mean percentage of symptomatic kernels per spike) were assessed for each inoculation/control window and entire micro plots. A head symptoms index was calculated by the formula *Head symptoms index = Disease incidence × Disease severity/100* and is represented in percent.

Micro plots were manually harvested and threshed at full grain maturity by the use of a stand-alone combine harvester. Barley heads grown in the inoculation/control windows were manually harvested and threshed. Therefore, up to 30 randomly picked mature heads (GS 99) were sampled for DNA extraction and quantification of *Fusarium* spp. DNA content from threshed grain material and stored under ambient and dry conditions.

### 5.8. Isolation of Genomic DNA from Mature Grain Material

For extraction of genomic DNA, a batch of 80 g barley grain was grinded to fine flour using a laboratory grinder. DNA extraction was conducted according to the instruction for isolating maize DNA by the European Community Reference Laboratories [79] with minor modification published by [44]. Quantity of isolated genomic DNA was determined with a Nanodrop ND-1000 spectrophotometer (Thermo Scientific) and diluted with sterile double distilled water to a final concentration of 20 ng/µL and stored at 4 °C until further processing.

### 5.9. Quantification of Fusarium spp. DNA

Genomic fungal DNA in mature grain was detected by quantitative polymerase chain reaction (qPCR) based on the protocol of [55] as reproduced by [44]. PCR amplifications were carried out with an AriaMx Real-time PCR System (Agilent Technologies Inc., Santa Clara, CA, USA). Therefore, barley and fungal DNA was measured in duplicates. *Fusarium* spp. DNA was normalized to barley DNA contents and is presented as picogram fungal DNA per nanogram barley DNA (pg fungal DNA/ng barley DNA).

### 5.10. Quantification of Multiple Fusarium spp. Toxins

Prior to LC-MS/MS analysis, sample preparation was performed as previously described by [67]. In brief, 1 g of the ground sample was spiked with internal standards in amounts adjusted to the expected contents of the respective analytes to fall in the given calibration range. After evaporation of the solvent overnight, 10 mL of acetonitrile/water (84/16, *v/v*) was added and extracted on a laboratory shaker at 325 rpm for 2 h. The sample was filtered, and 4 mL of the filtrate was applied on a Bond Elut Mycotoxin cartridge (500 mg, 3 mL) (Agilent Technologies, Santa Clara, CA, USA). The eluate was collected by applying vacuum, evaporated to dryness, reconstituted in 200 µL ACN/water (1/1, *v/v*) and then membrane filtered (0.22 µm). LC-MS/MS analysis was performed next. Starch (1 g) was selected as the blank matrix and spiked with 6–8 different concentrations of ZEA (0.5–30 µg/kg) and NIV (7.5–500 µg/kg) for matrix-matched calibration. FUSX could not be detected in any of the analysed samples and was therefore not considered in matrix-matched calibration. After solvent evaporation, the above-described sample preparation was performed.

For LC-MS/MS measurements, chromatographic separation was performed on a Shimadzu Nexera X2 UHPLC system (Shimadzu, Kyoto, Japan) using a Shim-pack Velox PFPP column (10 × 2.1 mm, 2.7 µm, Shimadzu, Duisburg, Germany) with a Shim-pack Velox PFPP guard-column (5 × 2.1 mm, Shimadzu, Duisburg, Germany) at 30 °C; the binary gradients are listed in the Table S7 and the injection volumes were 5 µL. The LC was interfaced with a Shimadzu 8050 triple quadrupole mass spectrometer (Shimadzu Corporation, Kyoto, Japan) using electrospray ionization (ESI) and the scheduled multiple reaction monitoring (MRM) mode for MS/MS measurements at the conditions detailed in Supplementary Material (Tables S7 and S8). It operated in the negative ESI mode for the analytes NIV, DON3G, DON and ZEA and in the positive ESI mode for the analytes 3ADON, 15ADON, FUSX, HT2-toxin, T2-toxin, ENN B, B1, A, A1 and BEA. For data acquisition and data analysis, the LabSolutions Software (Shimadzu, Kyoto, Japan) was used. For calibration and quantitation, response functions were obtained using linear regression by plotting peak area ratios [A(A)/A(IS)] against molar ratios [n(A)/n(IS)]. For 15ADON, ^13^[C_17_]-3ADON was used as the internal standard, and the enniatins were calculated using ^15^[N_3_]-Enniatin A1 as the internal standard. For analytes without a stable isotope-labelled standard (NIV, ZEA and fusarenone X), matrix-matched calibration was performed using a mycotoxin-free starch as blank matrix. After LC-MS/MS measurements, matrix-matched calibration curves were obtained by plotting peak areas against concentrations and performing linear regression. Linearity was confirmed for all analytes by applying Mandel’s fitting test [80].

### 5.11. Calculation of Disease Severity Ranking

To balance season effects of FHB severity and to evaluate FHB resistance of each genotype, a ranking list was calculated as described by [56]. A mean ranking for DNA contents in mature barley heads and a total mean rank were calculated for each genotype and year. Therefore, each single value was genotype-wise transcribed to explicit ranks per year (minimum rank: 1; maximum rank 17), then averaged over years and sorted in an ascending order. Low DNA contents were equal to low ranks and indicate resistance; high DNA contents were equal to high ranks and indicate susceptibility. Shared single DNA contents were scored with same ranks. This method was similarly used to calculate disease severity rankings from data on mycotoxin contents in mature barley grain.

## Data Availability

Raw data, all figures, supplemental figures, supplemental tables and the corresponding data tables are online available at: https://doi.org/10.6084/m9.figshare.c.5798849 (accessed on 20 December 2021).

## Supplementary Materials

The following supporting information can be downloaded at: https://www.mdpi.com/article/10.3390/toxins14020125/s1. References [81,82,83,84,85,86] are cited in the Supplementary Materials. Table S1: Linear regression data for correlations of *Fusarium* spp. DNA contents and associated mycotoxins* detected in mature barley grain of 17 barley genotypes grown in plots inoculated with bruised grain material (*F. culmorum* or *F. avenaceum*) in seasons 2018, 2019 and 2020, Table S2: Sum of temperature in two meters above ground according to dates of anthesis between 2016 and 2020 and calculated means two and one week before anthesis and one, two, three and four weeks post anthesis, Table S3: Recorded sum of precipitation one meter above ground according to dates of anthesis between 2016 and 2020 and calculated means two and one week before anthesis and one, two, three and four weeks post anthesis, Table S4: Recorded mean relative air humidity two meters above ground according to dates of anthesis between 2016 and 2020 and calculated means two and one week before anthesis and one, two, three and four weeks post anthesis, Table S5: Linear regression data for correlations of plant height and mycotoxin* contents detected in mature barley grain of 17 barley genotypes grown in plots inoculated with bruised grain material (*F. culmorum* or *F. avenaceum*) in seasons 2019 and 2020, Table S6: Dates of sowing, growth stages (GS), harvest and number of tested genotypes in field trials and in seasons between 2016 and 2020, Table S7: Chromatographic and ion source parameters of LC-MS/MS for toxin analysis, Table S8: List of fragment ions and retention times (Rt) of the analysed mycotoxins and their corresponding optimized collision energies (CE) and voltages. Unlabelled reference compounds (deoxynivalenol-3-glucoside (D3G), 15-acetyl-deoxynivalenol (15-ADON), T2-toxin) and some labelled standards (^13^[C_15_]-DON, ^13^[C_17_]-3-ADON,^13^[C_22_]-HT2-toxin and ^13^[C_21_]-D3G) were purchased from Biopure (Tulln, Austria). DON, 3-acetyldeoxynivalenol (3-ADON) as well as fusarenone X (FUSX) were obtained from Coring System Diagnostix (Gernsheim, Germany). HT2-toxin and Zearalenone (ZEA) were purchased from Sigma Aldrich (Missouri, USA). Nivalenol (NIV), enniatins A and B were obtained from Cayman Chemicals (Michigan, USA), enniatins A1 and B1 from Enzo Life Sciences (Lörrach, Germany) and Beauvericin (BEA) from AnaSpec (San Jose, USA). ^13^[C_4_]-T2-toxin, ^15^[N_3_]-Enniatin A1 and ^15^[N_3_]-BEA were synthesized in our laboratory as reported previously [1,2], Figure S1: FHB resistance towards *F. culmorum* according to DNA contents and mean ranks in field trials between 2016 and 2020, Figure S2: FHB resistance towards *F. avenaceum* according to DNA contents and mean ranks in field trials between 2016 and 2020, Figure S3: Hierarchical clustering for genetic relationship between 17 spring barley genotypes, Figure S4: *Fusarium* infestation of 17 barley genotypes co-inoculated with bruised grain colonized with *F. culmorum* and *F. avenaceum* in micro plots in 2017 and 2018, Figure S5: Relations between mean plant height and mean *F. culmorum* or mean *F. avenaceum* DNA contents in mature barley heads in field trials between 2019 and 2020, Figure S6: Mean *Fusarium* spp. DNA contents according to bruised grain and spray inoculations in field trials between 2019 and 2020, Figure S7: Relations between mean *F. culmorum* or *F. avenaceum* DNA contents and respective head symptoms in field trials, Figure S8: Head symptoms index according to assessed head symptoms in inoculated and control plots, Figure S9: Recorded weather conditions in seasons between 2016 and 2020 according to date of anthesis, Figure S10: Adjusted R^2^ and respective *p* values of genotype-wise multiple linear regression models for the two weeks before and four weeks after anthesis relative to flowering date in the seasons 2016 to 2020, Figure S11: Ninety-five percent confidence intervals for each regression coefficient (β_0,_ β_1,_ β_2_ and β_3_) for the calculated MLR model, Figure S12: Ninety-five percent confidence intervals for each regression coefficient (β_0,_ β_1,_ β_2_ and β_3_) for the calculated MLR model, Figure S13: Ninety-five percent confidence intervals for each regression coefficient (β_0,_ β_1,_ β_2_ and β_3_) for the calculated MLR model, Figure S14: Ninety-five percent confidence intervals for each regression coefficient (β_0,_ β_1,_ β_2_ and β_3_) for the calculated MLR model, Figure S15: Ninety-five percent confidence intervals for each regression coefficient (β_0_, β_1_, β_2_ and β_3_) for the calculated MLR model, Figure S16: Ninety-five percent confidence intervals for each regression coefficient (β_0_, β_1_, β_2_ and β_3_) for the calculated MLR model.
