# Supplementary material for: Host Genotype and Weather Effects on Fusarium Head Blight Severity and Mycotoxin Load in Spring Barley
Source: Toxins (Basel). 2022 Feb 8;14(2):125. doi: 10.3390/toxins14020125 (PMC8879614; doi:10.3390/toxins14020125)
Supplement: Supplementary file 1 [file toxins-14-00125-s001.zip › toxins-1542330-supplementary.pdf]

Supplementary Materials: Host Genotype and Weather Effects on Fusarium Head Blight Severity and Mycotoxin Load in Spring Barley

Felix Hoheneder, Eva Maria Biehl, Katharina Hofer, Johannes Petermeier, Jennifer Groth, Markus Herz, Michael Rychlik, Michael Heß and Ralph Hückelhoven

Table S1. Linear regression data for correlations of *Fusarium* spp. DNA contents and associated mycotoxins* detected in mature barley grain of 17 barley genotypes grown in plots inoculated with bruised grain material (*F. culmorum* or *F. avenaceum*) in seasons 2018, 2019 and 2020.

Year	Inoculation	Mycotoxin	p Value	R^2	Linear Regression Equation
2018	*F. culmorum*	DON	0.002 (**)	0.4828	y = 13.16x + 45.57
2019	*F. culmorum*	DON	< 0.0001 (****)	0.7414	y = 16.23x − 17.16
2020	*F. culmorum*	DON	0.0498 (*)	0.2329	y = 58.90x + 553.9
2018	*F. culmorum*	NIV	0.614 (ns)	0.01738	y = -3.363x + 225.6
2019	*F. culmorum*	NIV	0.6426 (ns)	0.01473	y = 0.5338x + 12.35
2020	*F. culmorum*	NIV	0.3829 (ns)	0.05111	y = 7.067x + 28.80
2018	*F. culmorum*	DON3G	0.0191 (*)	0.3151	y = 3.509x + 14.20
2019	*F. culmorum*	DON3G	0.0002 (***)	0.6255	y = 4.991x − 4.735
2020	*F. culmorum*	DON3G	0.1173 (ns)	0.1555	y = 9.912x + 85.02
2018	*F. culmorum*	3ADON	0.0019 (**)	0.4865	y = 1.288x − 3.527
2019	*F. culmorum*	3ADON	0.0001 (***)	0.6387	y = 1.049x − 10.16
2020	*F. culmorum*	3ADON	0.0002 (***)	0.6199	y = 3.135x + 7.859
2018	*F. culmorum*	15ADON	0.0268 (*)	0.2865	y = 0.1484x − 0.4831
2019	*F. culmorum*	15ADON	0.9843 (ns)	0.00003	y = -0.002248x + 4.119
2020	*F. culmorum*	15ADON	0.1908 (ns)	0.1112	y = 1.264x + 13.14
2018	*F. culmorum*	ZEA	0.6078 (ns)	0.01799	y = 0.01380x + 0.2263
2019	*F. culmorum*	ZEA	no ZEA detected in 2019		
2020	*F. culmorum*	ZEA	0.5699 (ns)	0.022	y = 0.06419x + 0.9584
2018	*F. avenaceum*	ENN A	0.0779 (ns)	0.1927	y = 1.185x + 9.643
2019	*F. avenaceum*	ENN A	0.0002 (***)	0.6067	y = 2.076x − 6.186
2020	*F. avenaceum*	ENN A	0.7487 (ns)	0.007046	y = 0.2911x + 5.141
2018	*F. avenaceum*	ENN A1	0.01 (**)	0.3666	y = 11.41x + 60.38
2019	*F. avenaceum*	ENN A1	0.0003 (***)	0.5864	y = 25.42x − 73.95
2020	*F. avenaceum*	ENN A1	0.4409 (ns)	0.04011	y = 7.117x + 47.01
2018	*F. avenaceum*	ENN B	< 0.0001 (****)	0.8513	y = 28.65x − 94.26
2019	*F. avenaceum*	ENN B	0.0004 (***)	0.574	y = 202.6x − 380.0
2020	*F. avenaceum*	ENN B	0.0084 (**)	0.3797	y = 179.7x + 185.4
2018	*F. avenaceum*	ENN B1	< 0.0001 (****)	0.6677	y = 75.56x + 131.1
2019	*F. avenaceum*	ENN B1	0.0004 (***)	0.5735	y = 58.26x − 121.5
2020	*F. avenaceum*	ENN B1	0.0255 (*)	0.2907	y = 42.57x + 78.26
2018	*F. avenaceum*	BEA	0.1629 (ns)	0.1256	y = 0.2243x + 4.561

2019	*F. avenaceum*	BEA	0.0418 (*)	0.2482	y = 0.2253x + 0.03391
2020	*F. avenaceum*	BEA	0.0095 (**)	0.3702	y = 0.3500x + 0.4049
2018	*F. avenaceum*	sum of ENNs	< 0.0001 (****)	0.676	y = 116.8x + 106.9
2019	*F. avenaceum*	sum of ENNs	0.0004 (***)	0.5778	y = 288.4x − 581.6
2020	*F. avenaceum*	sum of ENNs	0.0136 (**)	0.3427	y = 229.7x + 315.8

* DON = deoxynivalenol; NIV = nivalenol; DON3G = deoxynivalenol-3-glucoside; 3ADON = 3-acetyl-deoxynivalenol; 15ADON = 15-acety-deoxynivalenol; ZEA = zearalenon; ENN A = enniatin A, ENN A1 = enniatin A1, ENN B = enniatin B; ENN B1 = enniatine B1; BEA = beauvericin; sum of enniatins = sum of ENN A, ENN A1, ENN B and ENN B1 contents. Statistically significant correlations are indicated by * $p < 0.05$, ** $p < 0.01$, *** $p < 0.001$ and **** $p < 0.0001$; ns = not significant.

Table S2. Sum of temperature in two meters above ground according to dates of anthesis between 2016 and 2020 and calculated means two and one week before anthesis and one, two, three and four weeks post anthesis. The data were collected by a weather station at the location in Freising. The data were accessed from the agro-meteorology web portal of the Bavarian State Institute for Agriculture [78]. Approximate dates of full anthesis (GS 65): 1st of July (2016), 15th of June (2017, 2018), 21st of June (2019), 7th of June (2020).

	**Sum of Temperature [°C]**					
**Time Period**	**2016**	**2017**	**2018**	**2019**	**2020**	**mean**
2nd week pre anthesis	117.6	114.3	138.8	119.9	83.3	**114.8**
1st week pre anthesis	132.3	120.8	127.7	138.0	104.6	**124.7**
1st week post anthesis	123.2	136.9	128.0	149.5	100.8	**127.7**
2nd week post anthesis	131.6	149.5	100.4	141.2	103.8	**125.3**
3rd week post anthesis	126.1	123.5	127.8	118.4	129.6	**125.1**
4th week post anthesis	138.7	145.5	119.8	116.8	129.8	**130.1**
**mean**	**128.3**	**131.8**	**123.7**	**130.6**	**108.7**	**124.6**

Table S3. Recorded sum of precipitation one meter above ground according to dates of anthesis between 2016 and 2020 and calculated means two and one week before anthesis and one, two, three and four weeks post anthesis. The data were collected by a weather station at the location in Freising. The data were accessed from the agro-meteorology web portal of the Bavarian State Institute for Agriculture [78]. Approximate dates of full anthesis (GS 65): 1st of July (2016), 15th of June (2017, 2018), 21st of June (2019), 7th of June (2020).

	**Sum of Precipitation [mm]**					
**Time Period**	**2016**	**2017**	**2018**	**2019**	**2020**	**mean**
2nd week pre	14.6	12.4	17.6	19.4	1.4	**13.1**

anthesis						
1st week pre anthesis	36.8	6.9	80.7	26.8	11.0	**32.4**
1st week post anthesis	1.4	3.7	0.0	33.1	25.4	**12.7**
2nd week post anthesis	59.5	18.0	24.4	9.5	78.5	**38.0**
3rd week post anthesis	8.0	16.0	1.1	14.4	21.5	**12.2**
4th week post anthesis	20.1	39.7	11.5	14.0	43.2	**25.7**
**mean**	**23.4**	**16.1**	**22.6**	**19.5**	**30.2**	**22.4**

Table S4. Recorded mean relative air humidity two meters above ground according to dates of anthesis between 2016 and 2020 and calculated means two and one week before anthesis and one, two, three and four weeks post anthesis. The data were collected by a weather station at the location in Freising. The data were accessed from the agro-meteorology web portal of the Bavarian State Institute for Agriculture [78]. Approximate dates of full anthesis (GS 65): 1st of July (2016), 15th of June (2017, 2018), 21st of June (2019), 7th of June (2020).

	Mean Relative Air Humidity [%]					
**Time Period**	**2016**	**2017**	**2018**	**2019**	**2020**	**mean**
2nd week pre anthesis	76.2	85.9	76.9	82.6	73.4	**79.0**
1st week pre anthesis	75.1	68.2	86.1	82.9	71.8	**76.8**
1st week post anthesis	69.6	64.0	75.4	82.5	86.8	**75.7**
2nd week post anthesis	74.2	69.4	75.8	65.1	93.1	**75.5**
3rd week post anthesis	73.4	69.8	72.4	68.8	74.1	**71.7**
4th week post anthesis	82.2	74.4	79.7	77.7	81.8	**79.2**
**mean**	**75.1**	**71.9**	**77.7**	**76.6**	**80.2**	**76.3**

Table S5. Linear regression data for correlations of plant height and mycotoxin* contents detected in mature barley grain of 17 barley genotypes grown in plots inoculated with bruised grain material (*F. culmorum* or *F. avenaceum*) in seasons 2019 and 2020.

**Year**	***Fusarium* spp. DNA**	**Mycotoxin**	***p* Value**	**R Square**	**Linear Regression Equation**
2019	*F culmorum*	DON	0.5284 (ns)	0.02702	y = -9.890x + 1003
2020	*F culmorum*	DON	0.765 (ns)	0.006138	y = 5.288x + 305.3
2019	*F culmorum*	NIV	0.1978 (ns)	0.108	y = -4.615x + 333.1
2020	*F culmorum*	NIV	0.2245 (ns)	0.09668	y = 5.374x − 355.9
2019	*F culmorum*	DON3G	0.4481 (ns)	0.03888	y = -3.973x + 371.5
2020	*F culmorum*	DON3G	0.9469 (ns)	0.000306	y = 0.2431x + 91.60
2019	*F culmorum*	3ADON	0.641 (ns)	0.01487	y = 0.5112x − 21.22
2020	*F culmorum*	3ADON	0.5163 (ns)	0.02862	y = -0.3724x + 43.58
2019	*F culmorum*	15ADON	0.3627 (ns)	0.0555	y = -0.3284x + 26.06
2020	*F culmorum*	15ADON	0.2037 (ns)	0.1053	y = 0.6804x − 34.64
2019	*F culmorum*	ZEA	no ZEA detected in 2019		
2020	*F culmorum*	ZEA	0.2627 (ns)	0.08281	Y = 0.06886x − 4.037
2019	*F. aveneaceum*	ENN A	0.2677 (ns)	0.08118	y = -0.4764x + 36.63
2020	*F. aveneaceum*	ENN A	0.6049 (ns)	0.01828	y = -0.1941x + 20.51
2019	*F. aveneaceum*	ENN A1	0.2586 (ns)	0.0842	y = -6.044x + 464.5
2020	*F. aveneaceum*	ENN A1	0.3345 (ns)	0.06217	y = -3.669x + 342.3

2019	*F. aveneaceum*	ENN B	0.1909 (ns)	0.1112	y = -55.94x + 4432
2020	*F. aveneaceum*	ENN B	0.0366 (*)	0.2599	y = -61.56x + 5314
2019	*F. aveneaceum*	ENN B1	0.2235 (ns)	0.09705	y = -15.04x + 1192
2020	*F. aveneaceum*	ENN B1	0.0512 (ns)	0.2304	y = -15.69x + 1376
2019	*F. aveneaceum*	BEA	0.908 (ns)	0.0009195	y = -0.008605x + 1.796
2020	*F. aveneaceum*	BEA	0.1264 (ns)	0.1487	y = -0.09182x + 8.291
2019	*F. aveneaceum*	sum of ENNs	0.2022 (ns)	0.106	y = -77,50x + 6125
2020	*F. aveneaceum*	sum of ENNs	0.0413 (*)	0.2493	y = -81.12x + 7053
2019 + 2020	*F culmorum*	DON	0.1706 (ns)	0.05784	y = 14.34x − 495.8
2019 + 2020	*F culmorum*	NIV	0.3556 (ns)	0.02673	y = 2.214x − 121.7
2019 + 2020	*F culmorum*	DON3G	0.785 (ns)	0.00236	y = -0.6569x + 154.2
2019 + 2020	*F culmorum*	3ADON	0.8938 (ns)	0.0005654	y = 0.06000x + 10.10
2019 + 2020	*F culmorum*	15ADON	0.0191 (*)	0.1601	y = 0.7143x − 40.47
2019 + 2020	*F. aveneaceum*	ENN A	0.5026 (ns)	0.01416	y = -0.1503x + 16.02
2019 + 2020	*F. aveneaceum*	ENN A1	0.2673 (ns)	0.0383	y = -2.769x + 260.0
2019 + 2020	*F. aveneaceum*	ENN B	0.0428 (*)	0.1221	y = -40.16x + 3543
2019 + 2020	*F. aveneaceum*	ENN B1	0.0744 (ns)	0.09612	y = -9.961x + 899.5
2019 + 2020	*F. aveneaceum*	BEA	0.3425 (ns)	0.02819	y = -0.03551x + 3.836
2019 + 2020	*F. aveneaceum*	sum of ENNs	0.0538 (ns)	0.1113	y = -53.04x + 4719

* DON = deoxynivalenol; NIV = nivalenol; DON3G = deoxynivalenol-3-glucoside; 3ADON = 3-acetyl-deoxynivalenol; 15ADON = 15-acety-deoxynivalenol; ZEA = zearalenon; ENN A = enniatin A, ENN A1 = enniatin A1, ENN B = enniatin B; ENN B1 = enniatine B1; BEA = beauvericin; sum of enniatins = sum of ENN A, ENN A1, ENN B and ENN B1 contents. Statistically significant correlations are indicated by * $p < 0.05$, ** $p < 0.01$, ns = not significant.

Table S6. Dates of sowing, growth stages (GS), harvest and number of tested genotypes in field trials and in seasons between 2016 and 2020.

	**Field Trial**				
	**2016**	**2017**	**2018**	**2019**	**2020**
**Plot Size**	**Micro Plots**	**Micro Plots**	**Micro Plots; Field Plots**	**Field Plots**	**Field Plots**
**Number of Cultivars**	**59**	**59**	**59; 59**	**17**	**17**
3rd week March		sowing		sowing	
4th week March					sowing
1st week April					
2nd week April			sowing		
3rd week April	sowing				
4th week April					
1st week May				GS 49	
2nd week May					
3rd week May					GS 31
4th week May					
1st week June		GS 32	GS 55-59		GS 49-51
2nd week June	GS 32			GS 51 - 55	GS 61-63
3rd week June	GS 37		GS 73-83	GS 63 - 67	GS 63-67
4th week June					GS 71-73
1st week July	GS 49-71		GS 85-87	GS 85-87	
2nd week July					GS 83-85
3rd week July					
4th week July					GS 90
1st week August					
2nd week August		GS 90			
3rd week August	GS 90				

Table S7. Chromatographic and ion source parameters of LC-MS/MS for toxin analysis.

Ionisation Mode	ESI Positive	ESI Negative
Mobile phase	(A) 0.1% formic acid<br>(B) methanol + 0.1% formic acid	(A) water<br>(B) acetonitrile
Flow rate [L/min]	0.4 mL/min	
Binary gradient	Started at 6% B for 2 min, raised linearly to 90% B within 14 min, raised further to 99% B during 2 min, then maintained at 99% B for 1.5 min. Next, the mobile phase returned to 6% within 1.5 min. The system was equilibrated for 2 min.	Started at 10% B for 2 min, raised linearly from 10 to 99% B during 4 min and was held at 99% B for 1.5 min. Next, the mobile phase returned to 10% B during the next 1.5 min and the system was equilibrated for 2 min.
Interface temperature / interface voltage	350 °C / 3 kV	340 °C / 4.5 kV
Heat block temperature	450 °C	430 °C
Desolvation temperature	150 °C	170 °C
Heating gas flow/drying gas flow / nebulizing gas flow	10 L/min / 10 L/min / 3 L/min	10 L/min / 10 L/min / 3 L/min
Collision induced dissociation gas pressure	265 kPa	230 kPa

Table S8. List of fragment ions and retention times (Rt) of the analysed mycotoxins and their corresponding optimized collision energies (CE) and voltages. Unlabelled reference compounds (deoxynivalenol-3-glucoside (D3G), 15-acetyl-deoxynivalenol (15-ADON), T2-toxin) and some labelled standards (^{13}[C$_{15}$]-DON, ^{13}[C$_{17}$]-3-ADON,^{13}[C$_{22}$]-HT2-toxin and ^{13}[C$_{21}$]-D3G) were purchased from Biopure (Tulln, Austria). DON, 3-acetyldeoxynivalenol (3-ADON) as well as fusarenone X (FUSX) were obtained from Coring System Diagnostix (Gernsheim, Germany). HT2-toxin and Zearalenone (ZEA) were purchased from Sigma Aldrich (Missouri, USA). Nivalenol (NIV), enniatins A and B were obtained from Cayman Chemicals (Michigan, USA), enniatins A1 and B1 from Enzo Life Sciences (Lörrach, Germany) and Beauvericin (BEA) from AnaSpec (San Jose, USA). ^{13}[C$_4$]-T2-toxin, ^{15}[N$_3$]-Enniatin A1 and ^{15}[N$_3$]-BEA were synthesized in our laboratory as reported previously [81,82].

Analyte	Rt [min]	ESI-Mode	Precursor Ion *m/z*	Product ion *m/z*	Q1 Pre Bias [V]	CE [V]	Q3 Pre Bias [V]
**Nivalenol**	1.07	-	311.20	281.20 [a]	20	13	30
				138.20 [b]	20	24	30
**D3G**	1.28	-	457.25	427.30 [a]	12	19	28
				247.25 [b]	12	20	24
**[^{13}C$_{21}$]-D3G**	1.28	-	478.25	447.30 [a]	12	19	28
				261.25 [b]	12	20	24
**DON**	1.47	-	295.30	265.20 [a]	10	14	10
				247.20 [b]	10	15	40
**[^{13}C$_{15}$]-DON**	1.47	-	310.30	279.20 [a]	10	14	10
				247.20 [b]	10	15	10
**ZEA**	5.19	-	317.15	175.10 [a]	24	25	16
				131.05 [b]	24	30	22
**3-ADON**	7.84	+	339.10	231.25 [a]	−16	−13	−26
				175.20 [b]	−16	−25	−20
**[^{13}C$_{17}$]-3 ADON**	7.84	+	356.10	245.25 [a]	−16	−13	−26
				186.00 [b]	−16	−25	−20
**15-ADON**	7.62	+	339.25	261.20 [a]	−10	−11	−30
				321.25 [b]	−10	−8	−6
**Fusarenone X**	5.61	+	355.10	175.20 [a]	−12	−22	−20
				137.20 [b]	−12	−26	−28
**HT2-Toxin-Na**	11.4	+	447.15	345.15 [a]	−22	−19	−18
				285.20 [b]	−22	−21	−20
**[^{13}C$_{22}$]-HT2-Na**	11.4	+	469.15	362,15 [a]	−22	−19	−18
				300.20 [b]	−22	−21	−20
**T2-Toxin-Na**	12.6	+	489.10	245.15 [a]	−26	−27	−29
				387.15 [b]	−14	−21	−22
**[^{13}C$_4$]-T2-Toxin-Na**	12.6	+	493.10	245.15 [a]	−26	−27	−29
				391.15 [b]	−14	−21	−22
**Enn B**	15.8	+	640.75	196.25 [a]	−18	−25	−22
				214.25 [b]	−18	−25	−16

**Enn B1**	16.0	+	654.30	196.25 [a]	−34	−26	−23
				210.25 [b]	−32	−24	−24
**Enn A1**	16.2	+	668.70	210.25 [a]	−18	−24	−16
				100.20 [b]	−18	−60	−20
**Enn A**	16.4	+	682.70	210.20 [a]	−12	−25	−16
				100.15 [b]	−12	−55	−20
**[$^{15}N_3$]-Enn A1**	16.2	+	671.70	211.25 [a]	−18	−24	−16
				101.20 [b]	−18	−60	−20
**BEA**	16.3	+	784.55	134.20 [a]	−22	−59	−26
				244.25 [b]	−22	−32	−28
**[$^{15}N_3$]-BEA**	16.3	+	787.55	135.20 [a]	−22	−59	−26
				245.25 [b]	−22	−32	−28

[a] Quantifier; [b] Qualifier

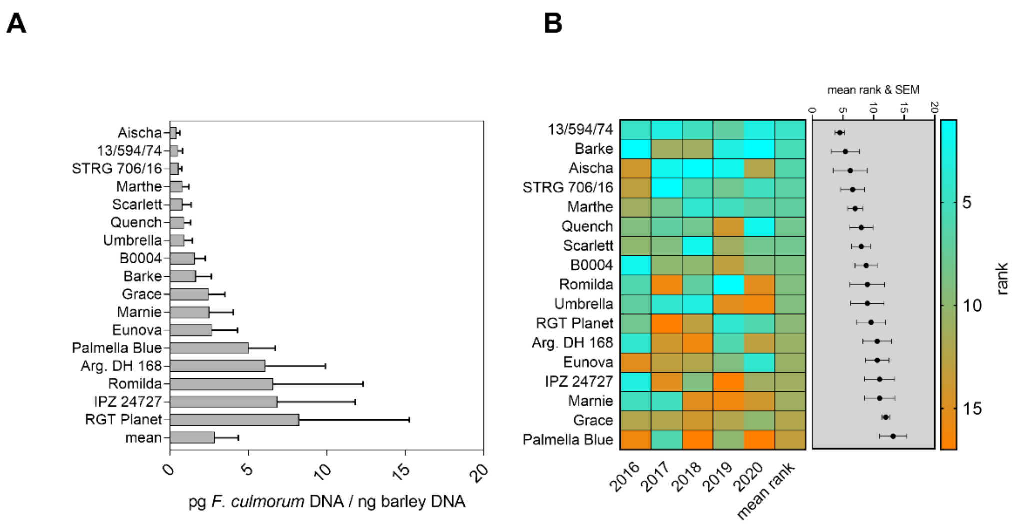

Figure S1. FHB resistance towards *F. culmorum* according to DNA contents and mean ranks in field trials between 2016 and 2020. The bar graphs represent mean *F. culmorum* DNA per genotype and total mean in non-inoculated sub plots in pg *F. culmorum* DNA/ng barley DNA (**A**). The heat map represents scored ranks according to detected *F. culmorum* DNA in mature barley heads in non-inoculated sub plots. Mean ranks of individual genotypes and the respective standard error of the mean are presented in the grey shaded box (**B**). Low ranks indicate high FHB resistance; high ranks indicate low FHB resistance. Error bars indicate standard error of the mean.

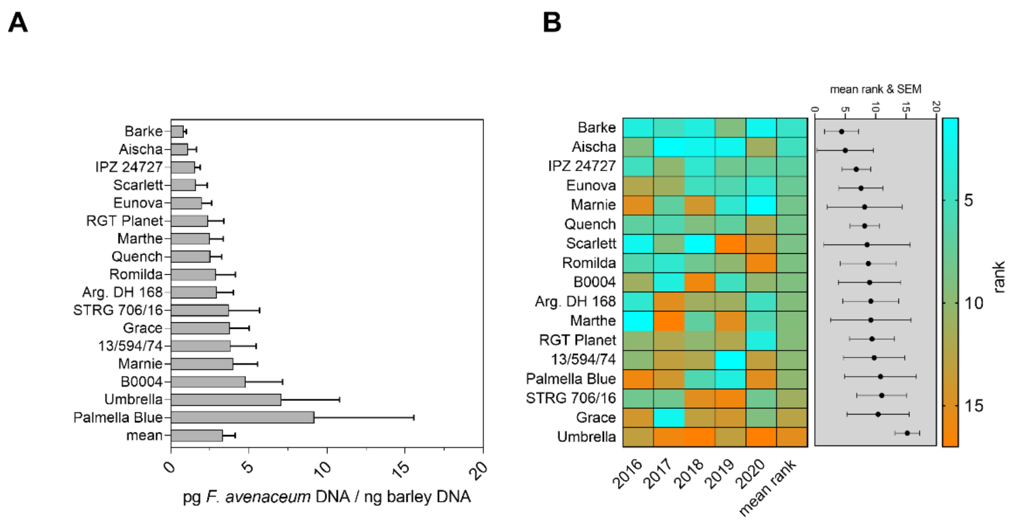

Figure S2. FHB resistance towards *F. avenaceum* according to DNA contents and mean ranks in field trials between 2016 and 2020. The bar graphs represent mean *F. avenaceum* DNA per genotype and total mean in non-inoculated sub plots in pg *F. avenaceum* DNA/ng barley DNA (**A**). The heat map represents scored ranks according to detected *F. avenaceum* DNA in mature barley heads in non-inoculated sub plots. Mean ranks of individual genotypes and the respective standard error of the mean are presented in the grey shaded box (**B**). Low ranks indicate high FHB resistance; high ranks indicate low FHB resistance. Error bars indicate standard error of the mean.

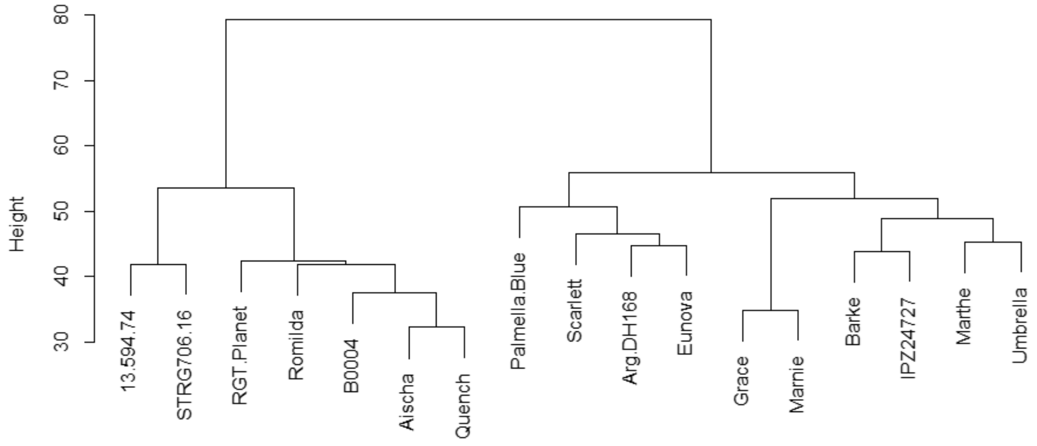

Figure S3. Hierarchical clustering for genetic relationship between 17 spring barley genotypes. The dendrogram was generated according to Ward's minimum variance method [83] using SNP marker data from a 9k iSelect SNP chip [84] from a previous project (Institute of Crop Science and Plant Breeding, Bavarian State Institute of Agriculture). Data analysis was performed with the R package Synbreed [85] in R [86]. Redundant markers and SNPs with an error value above 10 percent were excluded from the analysis resulting in 1299 SNP markers. Height represents genetic distance between individual spring barley genotypes.

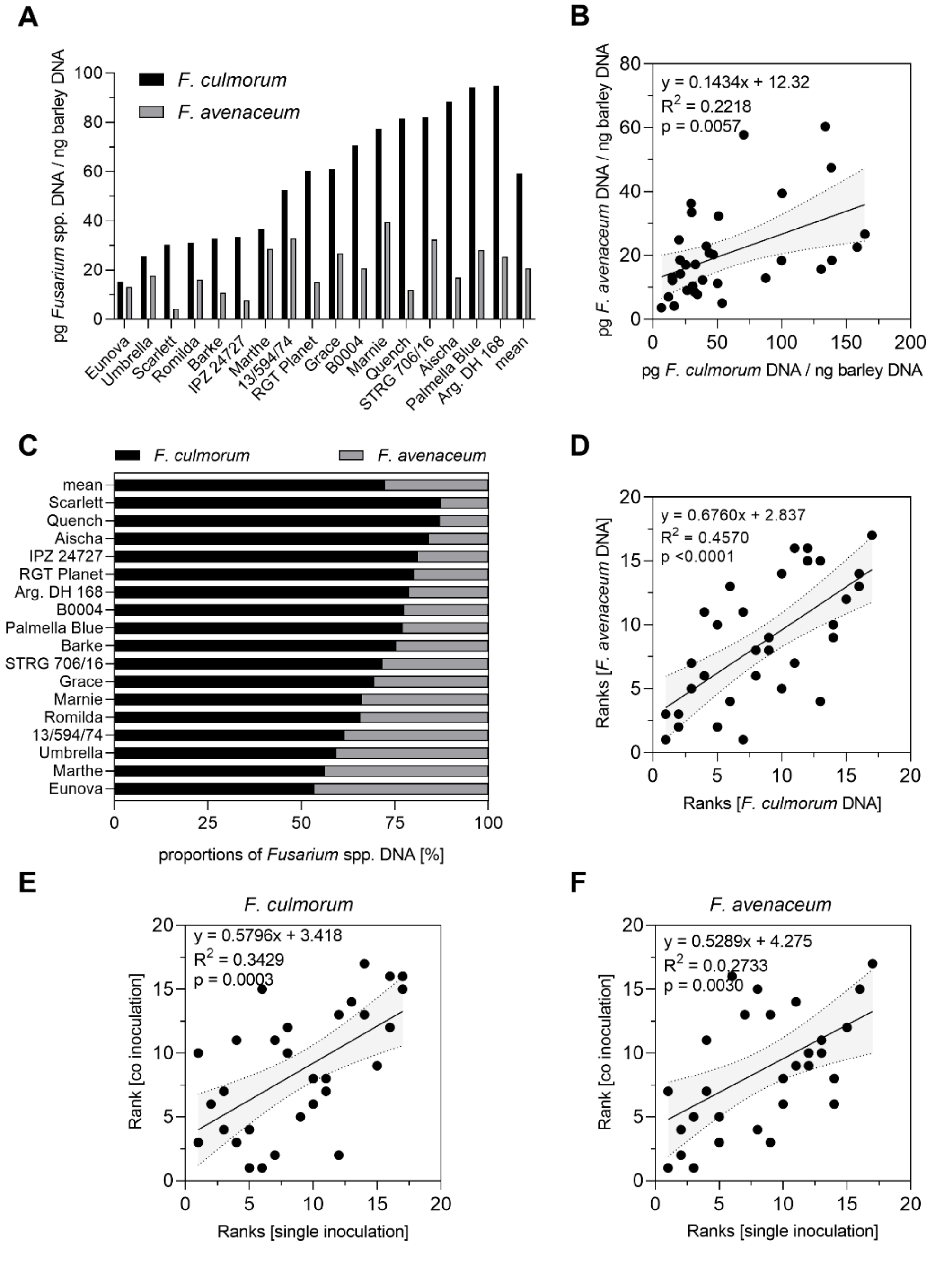

Figure S4. *Fusarium* infestation of 17 barley genotypes co-inoculated with bruised grain colonized with *F. culmorum* and *F. avenaceum* in micro plots in 2017 and 2018. Bar graphs indicate mean DNA contents of *F. culmorum* and *F. avenaceum*, respectively, in mature barley heads presented in pg *F.* spp. DNA/ng barley DNA (**A**). (**B**) Represents correlation between mean fungal DNA contents of *F. culmorum* and *F. avenaceum,* and (**C**) shows mean proportions of *Fusarium* spp. DNA contents in co-inoculated barley heads. (**D**) Represents mean genotype ranks according to *F.* spp. DNA contents in co-inoculated grains. (**E**) and (**F**) Show correlations between genotype ranks according to *F.* spp. DNA contents in co-inoculated and single inoculated barley grain. Statistical analysis: Sidak's multiple comparison test following 2-way ANOVA for comparison of genotype means. Error bars indicate

standard error of the mean; simple linear regression; *p* value indicates significance of the slope from zero. Grey shaded areas represent 95% confidence bands of the best-fit line.

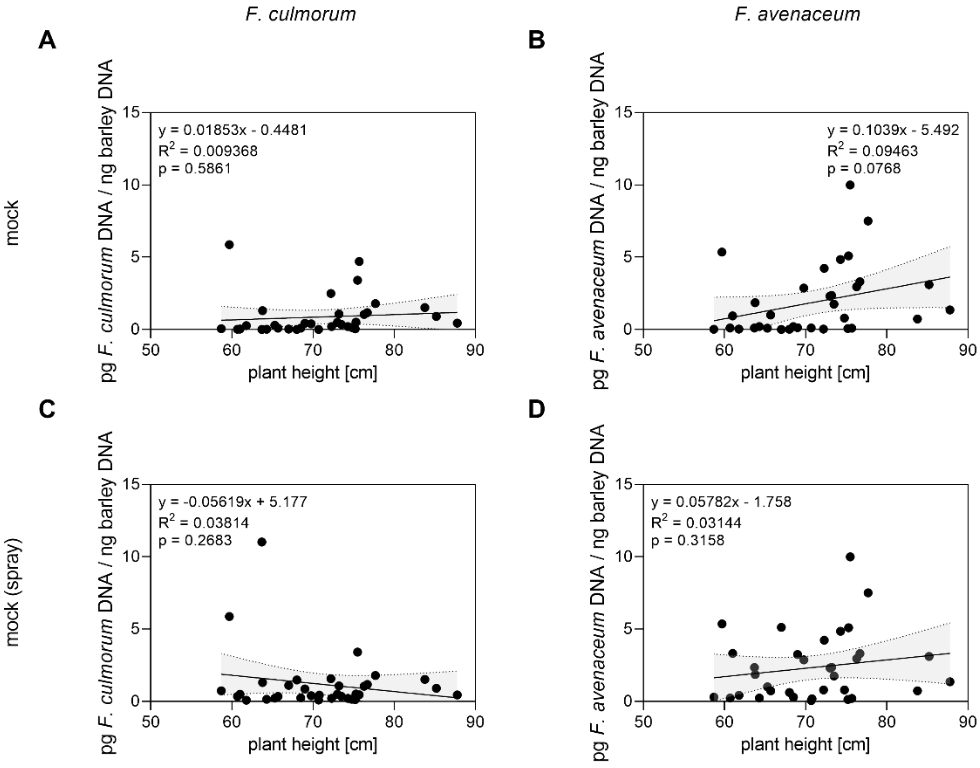

Figure S5. Relations between mean plant height and mean *F. culmorum* or mean *F. avenaceum* DNA contents in mature barley heads in field trials between 2019 and 2020. Data points represent data of 17 genotypes in naturally infected (**A**,**B**) and in mock-sprayed (**C**,**D**) plots, respectively, and mean plant height of each genotype recorded in 2019 and 2020. Data analysis: Simple linear regression; *p* value indicates significance of the slope from zero. Grey shaded areas represent 95% confidence bands of the best-fit line.

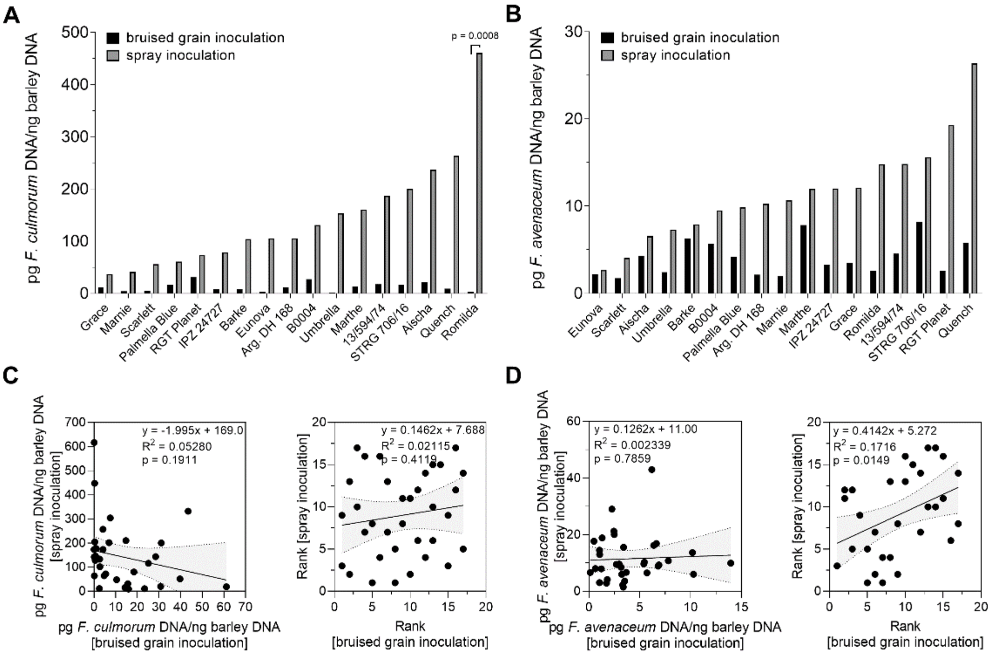

Figure S6. Mean *Fusarium* spp. DNA contents according to bruised grain and spray inoculations in field trials between 2019 and 2020. (**A**) Represents means of *F. culmorum* DNA/ng barley DNA, (**B**) represents means of *F. avenaceum* DNA/ng barley DNA of 17 genotypes, and (**C**) presents relations between *F. culmorum* contents or respective resistance ranks according to inoculation methods. (**D**) presents relations between *F. avenaceum* contents or respective resistance ranks according to inoculation methods. Error bars indicate standard error of the mean. Statistical analysis: 2-way ANOVA with Sidak's multiple comparisons of means; simple linear regression, *p* value indicates significance of the slope from zero. Grey shaded areas represent 95% confidence bands of the best-fit line.

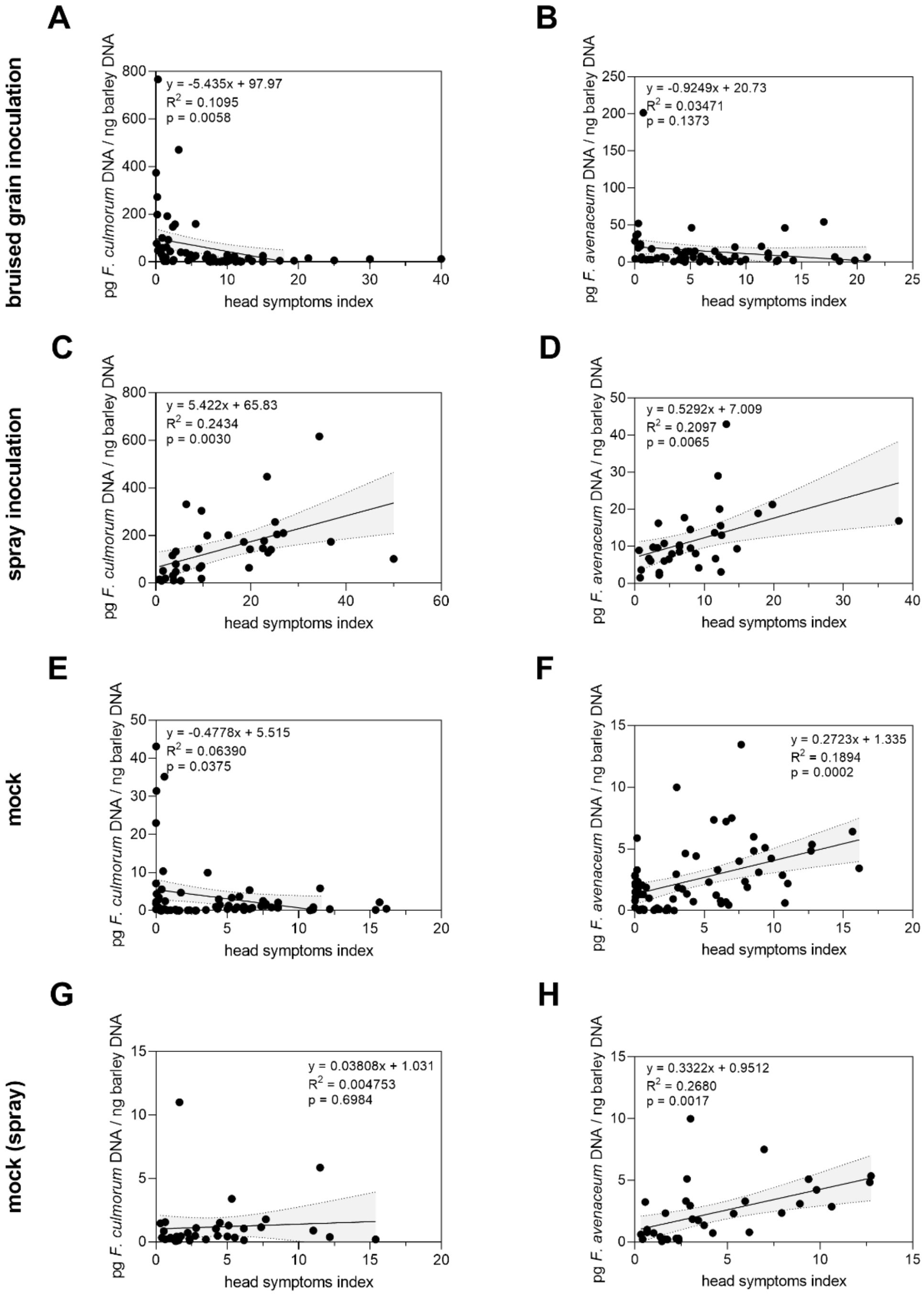

Figure S7. Relations between mean *F. culmorum* or *F. avenaceum* DNA contents and respective head symptoms in field trials. Data points represent calculated means of 17 genotypes in bruised grain inoculated (2017–2020) (**A**,**B**), in spray inoculated (2019–2020) (**C**,**D**), in non-inoculated (2017–2020), (**E**,**F**) and mock-sprayed (2019–2020) plots (**G**,**F**), respectively. Spray inoculations were exclusively conducted in 2019 and 2020. Percentage of symptoms per head (severity) and percentage of symptomatic heads (incidence) were separately assessed to calculate a head symptoms index (head symptoms index = severity × incidence / 100) and is represented as total mean. Data analysis: Simple linear regression; *p* value indicates significance of the slope from zero. Grey shaded areas represent 95% confidence bands of the best-fit line.

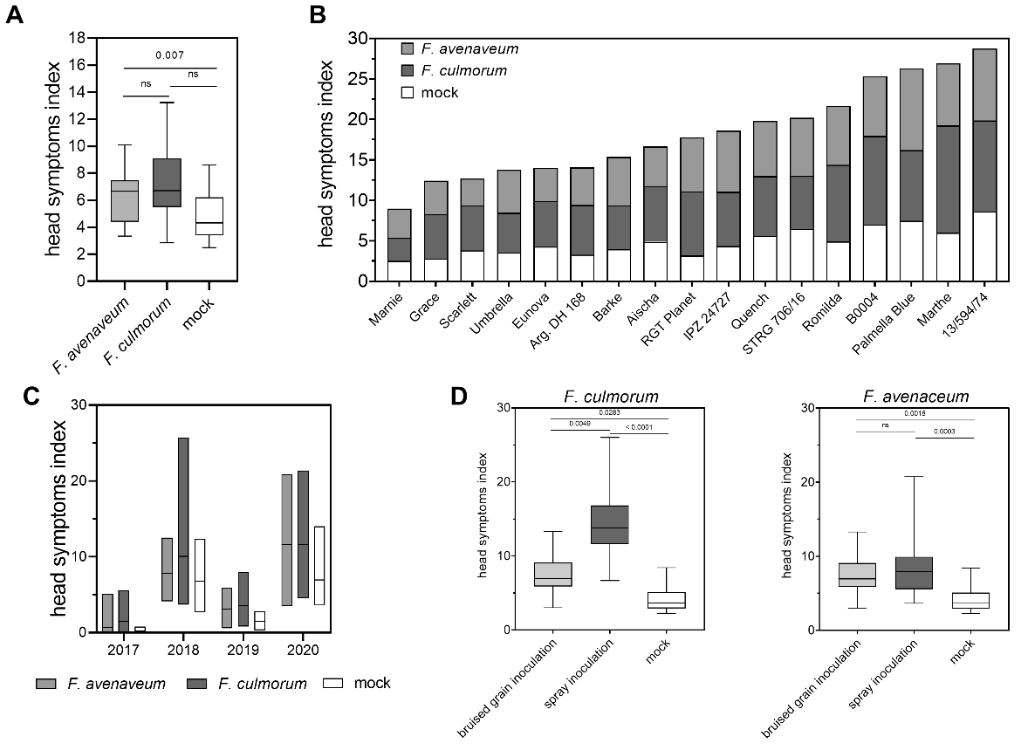

Figure S8. Head symptoms index according to assessed head symptoms in inoculated and control plots. (**A**-**C**) Show mean head symptoms indices of barley genotypes inoculated with *Fusarium culmorum* or *F. avenaceum* colonized bruised grain material and non-inoculated plots in seasons between 2017 and 2020. (**A**) Indicates comparison of mean head symptoms indices across 17 barley genotypes. (**B**) Shows genotype specific mean of head symptoms indices. (**C**) Shows mean head symptoms indices of differently inoculated plots across 17 barley genotypes. (**D**) Shows a comparison of mean head symptoms indices of differently inoculated and control plots across 17 barley genotypes in seasons 2019 and 2020. Statistical analysis: Kruskal–Wallis test with Dunn's multiple comparison.

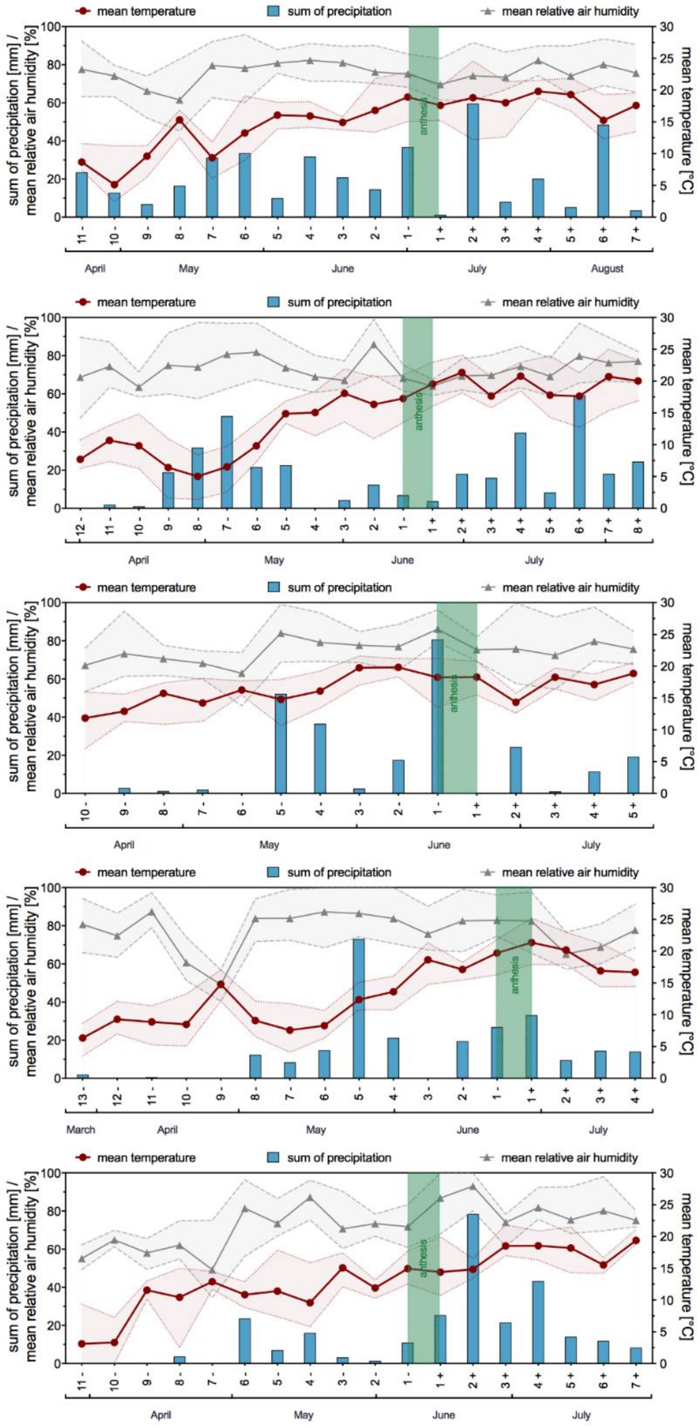

Figure S9. Recorded weather conditions in seasons between 2016 and 2020 according to date of anthesis. Each graph shows weekly sum of precipitation, weekly mean temperature and weekly mean relative air humidity 2 m above ground in the weeks pre and post anthesis. Blue bars show weekly sum of precipitation. Grey-coloured background indicates range of weekly mean of minimum and maximum temperatures, respectively. Red-coloured background indicates range of weekly mean of minimum and maximum relative air humidity, respectively. Green-coloured bars indicate approximate time of anthesis.

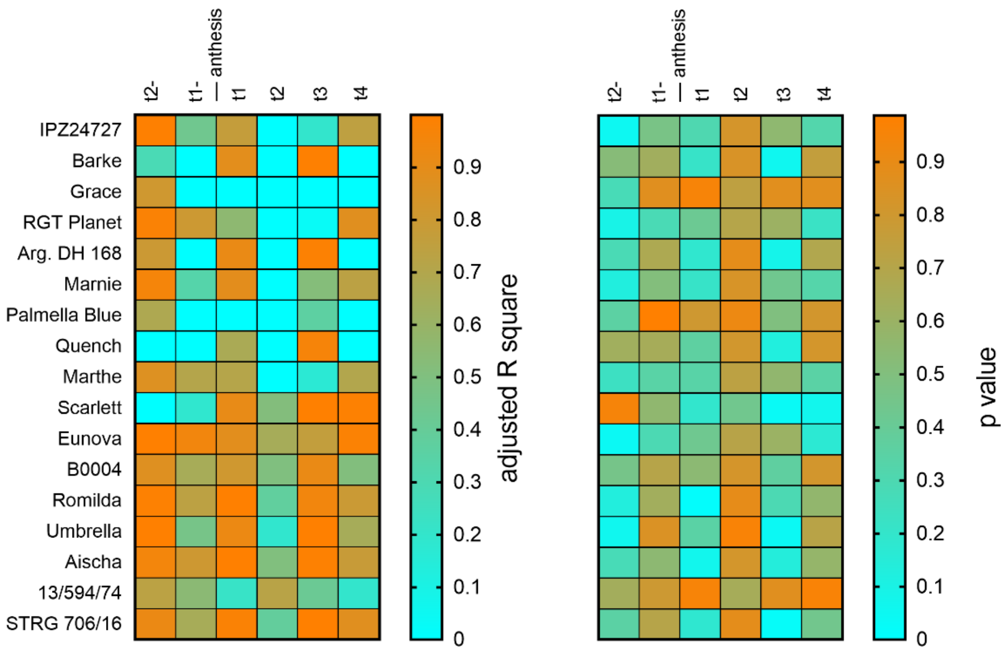

Figure S10. Adjusted R^2 and respective *p* values of genotype-wise multiple linear regression models for the two weeks before and four weeks after anthesis relative to flowering date in the seasons 2016 to 2020. The model Equation (1) was used to calculate multiple linear regressions to determine effects of weather conditions before and after anthesis on content of *F. avenaceum* DNA in mature barley heads.

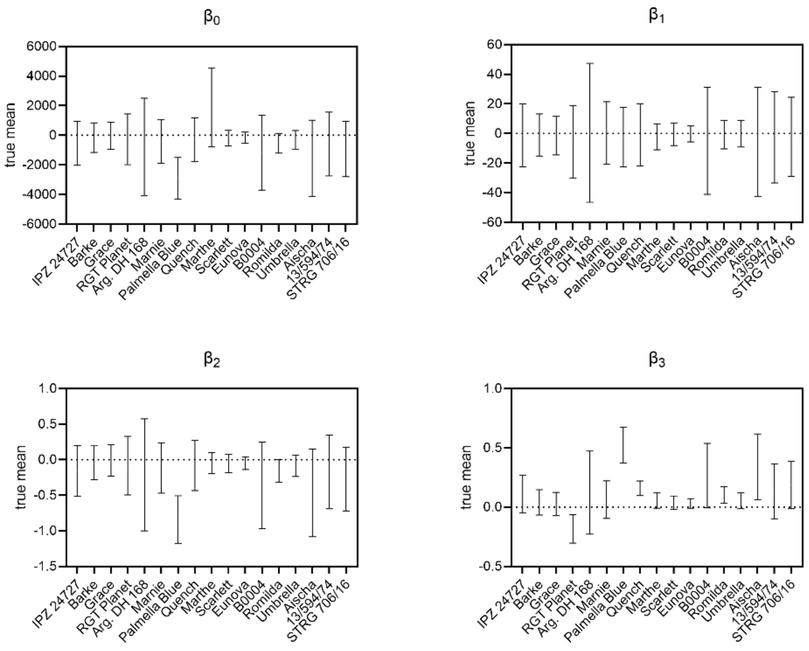

Figure S11. Ninety-five percent confidence intervals for each regression coefficient (β_0, β_1, β_2 and β_3) for the calculated MLR model. Input data are *F. culmorum* DNA contents in mature heads of 17 barley genotypes. Explanatory variables are sum of temperature (T_{SUM}), sum of precipitation (P_{SUM}) and average relative air humidity (RH_{AVG}) for the second week before anthesis. Confidence intervals represent variation of the respective regression coefficient and effect on the entire MLR model. Confidence intervals above or below zero indicate significant effects of the regression coefficient on the entire model.

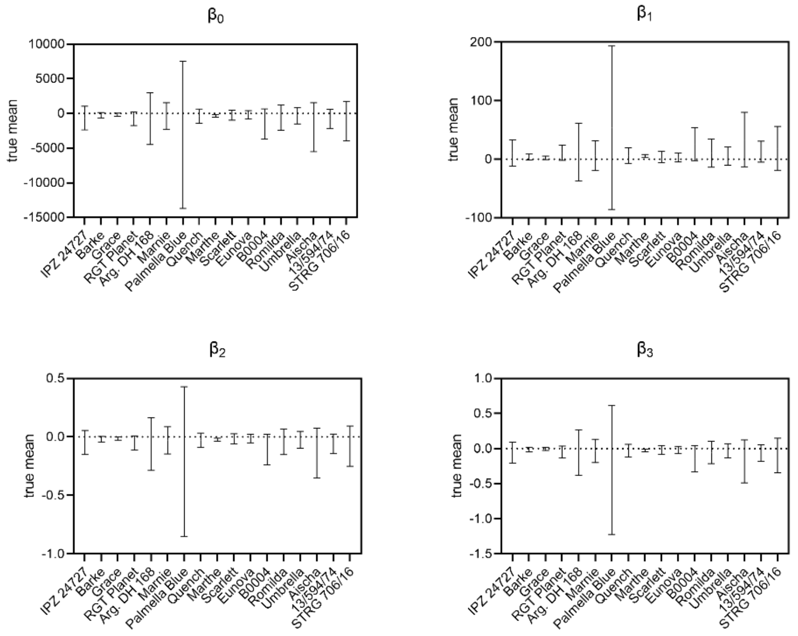

Figure S12. Ninety-five percent confidence intervals for each regression coefficient (β_0, β_1, β_2 and β_3) for the calculated MLR model. Input data are *F. culmorum* DNA contents in mature heads of 17 barley genotypes. Explanatory variables are sum of temperature (T_{SUM}), sum of precipitation (P_{SUM}) and average relative air humidity (RH_{AVG}) for the week before anthesis. Confidence intervals represent variation of the respective regression coefficient and effect on the entire MLR model. Confidence intervals above or below zero indicate significant effects of the regression coefficient on the entire model.

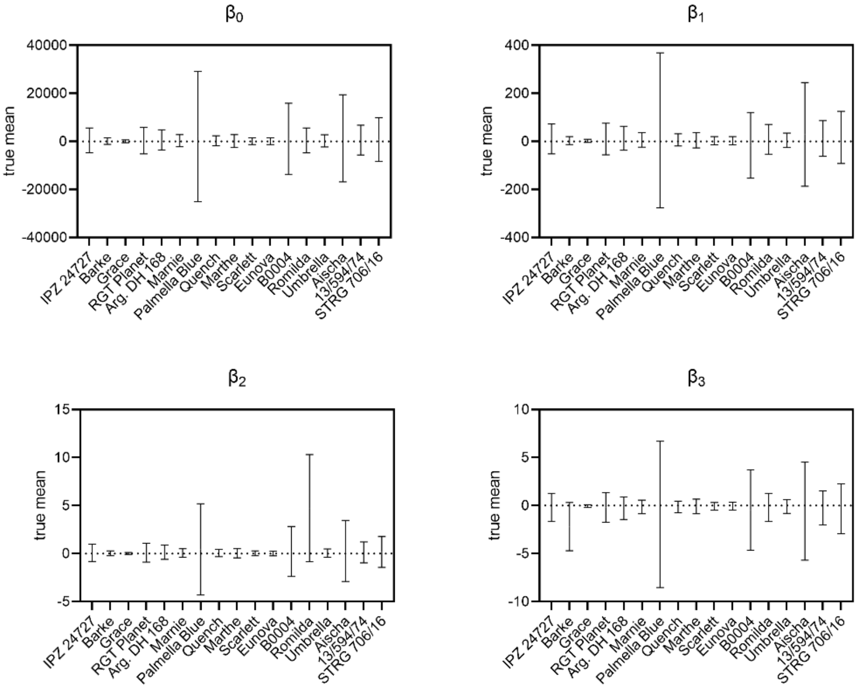

Figure S13. Ninety-five percent confidence intervals for each regression coefficient (β_0, β_1, β_2 and β_3) for the calculated MLR model. Input data are *F. culmorum* DNA contents in mature heads of 17 barley genotypes. Explanatory variables are sum of temperature (T_{SUM}), sum of precipitation (P_{SUM}) and average relative air humidity (RH_{AVG}) for the first week after anthesis. Confidence intervals represent variation of the respective regression coefficient and effect on the entire MLR model. Confidence intervals above or below zero indicate significant effects of the regression coefficient on the entire model.

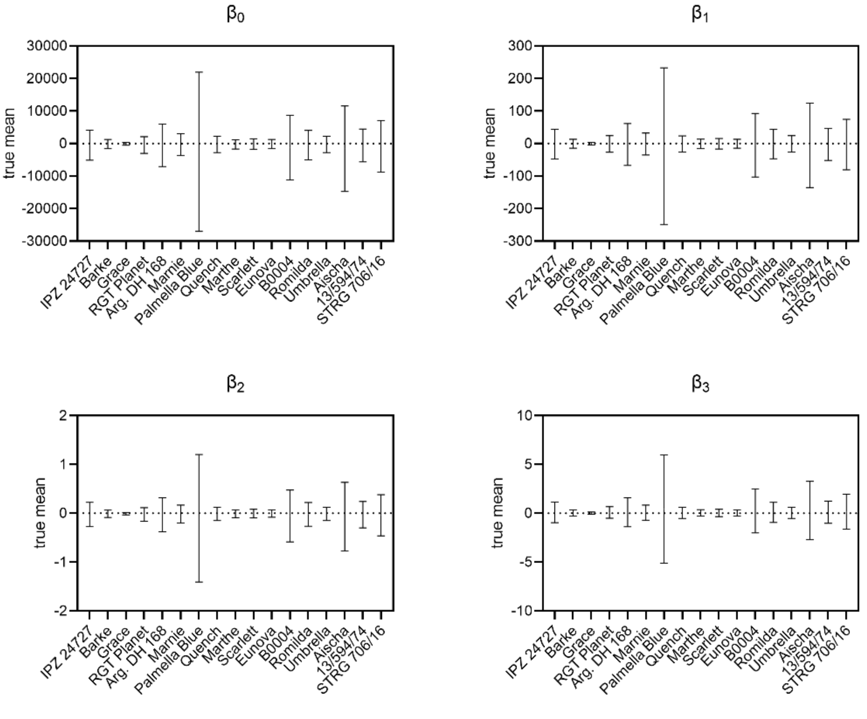

Figure S14. Ninety-five percent confidence intervals for each regression coefficient (β_0, β_1, β_2 and β_3) for the calculated MLR model. Input data are *F. culmorum* DNA contents in mature heads of 17 barley genotypes. Explanatory variables are sum of temperature (T_{SUM}), sum of precipitation (P_{SUM}) and average relative air humidity (RH_{AVG}) for the second week after anthesis. Confidence intervals represent variation of the respective regression coefficient and effect on the entire MLR model. Confidence intervals above or below zero indicate significant effects of the regression coefficient on the entire model.

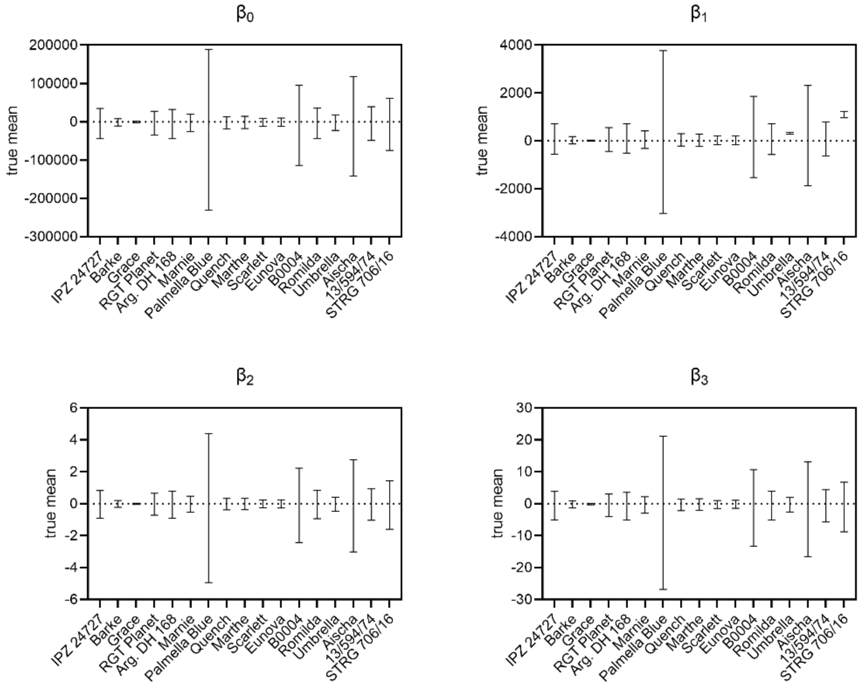

Figure S15. Ninety-five percent confidence intervals for each regression coefficient (β_0, β_1, β_2 and β_3) for the calculated MLR model. Input data are *F. culmorum* DNA contents in mature heads of 17 barley genotypes. Explanatory variables are sum of temperature (T_{SUM}), sum of precipitation (P_{SUM}) and average relative air humidity (RH_{AVG}) for the third week after anthesis. Confidence intervals represent variation of the respective regression coefficient and effect on the entire MLR model. Confidence intervals above or below zero indicate significant effects of the regression coefficient on the entire model.

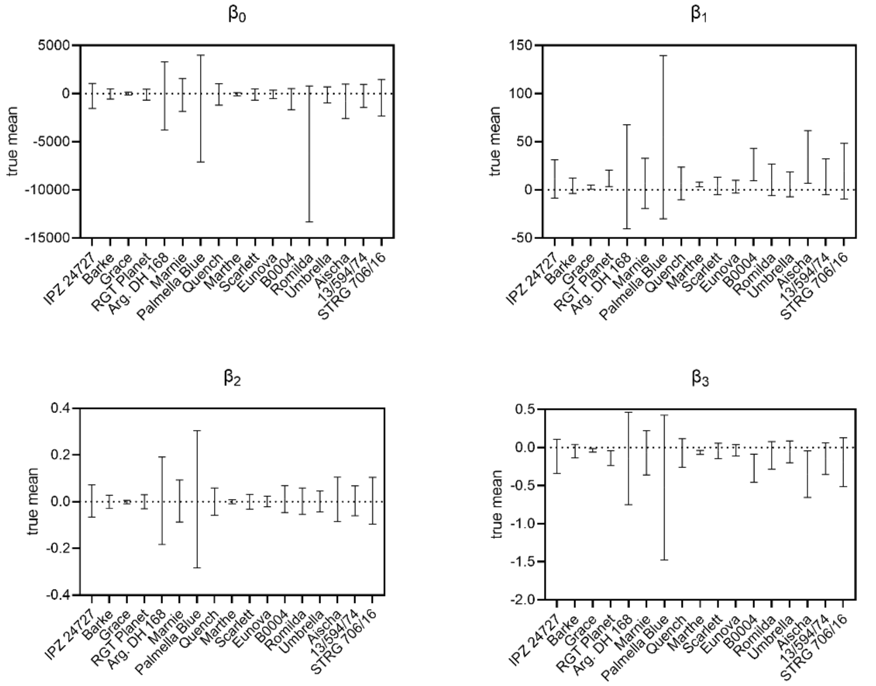

Figure S16. Ninety-five percent confidence intervals for each regression coefficient (β_0, β_1, β_2 and β_3) for the calculated MLR model. Input data are *F. culmorum* DNA contents in mature heads of 17 barley genotypes. Explanatory variables are sum of temperature (T_{SUM}), sum of precipitation (P_{SUM}) and average relative air humidity (RH_{AVG}) for the fourth week after anthesis. Confidence intervals represent variation of the respective regression coefficient and effect on the entire MLR model. Confidence intervals above or below zero indicate significant effects of the regression coefficient on the entire model.
